# Depression Aggravates Immune‐Mediated Hepatitis Through NLRP3 Overactivation Induced by Intestinal Microbiota

**DOI:** 10.1002/cns.70743

**Published:** 2026-01-08

**Authors:** Simin Zhou, Liping Guo, Nian Chen, Haifeng Liu, Xin Liu, Jiwen Li, Shijing Dong, Jiangpeng Liu, Xiaoyi Wang, Ying Ran, Man Liu, Hongyu Chu, Yanni Li, Hui Yang, Jingwen Zhao, Lu Zhou

**Affiliations:** ^1^ Department of Gastroenterology and Hepatology Tianjin Medical University General Hospital Tianjin China

**Keywords:** autoimmune hepatitis (AIH), bacterial translocation, chronic unpredictable mild stress (CUMS), depression, fecal microbiota transplantation (FMT), intestinal microbiota dysbiosis, *Lactococcus formosensis*, NLR family pyrin domain containing 3 (NLRP3)

## Abstract

**Background:**

Depression is associated with adverse effects in patients with autoimmune hepatitis (AIH). However, the underlying mechanism remains unclear. This study explores the impact of depression and related intestinal microbiota on immune‐mediated hepatitis.

**Methods:**

We assessed depression in 260 AIH patients receiving 2‐year standardized treatment and 173 healthy controls. In mice, depressive‐like behaviors were induced by chronic unpredictable mild stress (CUMS), and immune‐mediated hepatitis was induced by intravenous injection of concanavalin A (ConA). Fecal microbiota transplantation (FMT) was performed using samples from patients with major depressive disorder (MDD) and controls.

**Results:**

Depression was common in patients with AIH (106/260, 40.8%) and was associated with cirrhosis. Compared with nondepressed AIH patients, those with depression showed exacerbated intestinal barrier dysfunction and hepatic NLR family pyrin domain containing 3 (NLRP3) inflammasome overactivation. In the ConA‐induced hepatitis model, CUMS exposure aggravated these abnormalities, which were then attenuated by mirtazapine. Furthermore, mice colonized with MDD microbiota exhibited greater intestinal barrier disruption and hepatic NLRP3 inflammasome overactivation than those colonized with control microbiota. Notably, gut‐derived *Lactococcus formosensis*, isolated from the livers of MDD microbiota‐colonized mice, could translocate to the liver and induce hepatic NLRP3 inflammasome overactivation. In addition, vaccination against *L. formosensis* prevented translocation and alleviated liver injury in monocolonized mice.

**Conclusion:**

Depression aggravates immune‐mediated hepatitis through disruption of intestinal barrier integrity and overactivation of hepatic NLRP3 inflammasome. Gut‐derived *L. formosensis* could translocate to the liver and induce liver injury in mice. This study provides the necessity of screening for depression in patients with AIH.

## Graphical abstract

Depression is common in AIH patients and is associated with an elevated cirrhosis risk. AIH patients with depression, mice colonized with MDD microbiota, and ConA‐treated mice subjected to CUMS exhibit intestinal barrier dysfunction and hepatic NLRP3 inflammasome overactivation. Gut‐derived *Lactococcus formosensis*, isolated from the livers of mice harboring MDD microbiota, play pivotal roles in the disruption of intestinal epithelial integrity and overactivation of hepatic NLRP3 inflammasome.

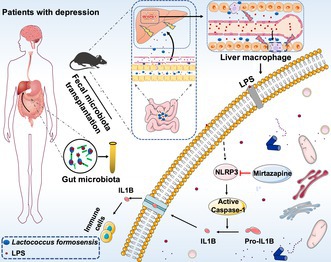

## 1 Introduction

Autoimmune hepatitis (AIH), characterized by the disruption of immune tolerance, is an immune‐mediated hepatitis with an increasing incidence [1]. A major depressive syndrome is five times more frequent in patients with AIH than in the general population [2]. Moreover, patients with other autoimmune diseases (AIDs) exhibit a significantly higher prevalence of depression than healthy controls [3, 4]. Depression increases the risk of nonadherence to standard therapy and relapse in patients with AIH [5], and is indeed an important risk factor for liver fibrosis [6, 7, 8]. Notably, patients with major depressive disorder (MDD) exhibit elevated frequencies of antinuclear antibody (ANA), thyroid gland antibody, and parietal cell antibody [9]. Similarly, another study demonstrated the potential role of T helper 17 cells in autoimmune disorders in patients with MDD [10], further supporting the existing evidence of autoimmune dysregulation in depression. Therefore, the role of depression in immune disorders during AIH progression warrants clinical attention.

Accumulating evidence suggests that depression drives inflammation and promotes immune activation [9, 10]. Macrophages play vital roles in maintaining immune homeostasis in the liver [11]. However, the persistent activation of hepatic macrophages results in pathological inflammation and fibrosis in chronic liver disease [12]. Meanwhile, liver‐resident macrophages can produce significant amounts of NLR family pyrin domain containing 3 (NLRP3) and interleukin 1 beta (IL1B) [13]. The NLRP3 inflammasome complex, which consists of NLRP3, pro‐caspase‐1 (proCASP1), and apoptosis‐associated speck‐like protein, can regulate the cleavage and activation of serine protease CASP1 and drive the production of pro‐inflammatory cytokine IL1B [14]. Several studies have indicated that the NLRP3 inflammasome is involved in the progression of depression [15, 16, 17]. NLRP3 activation is elevated in peripheral blood mononuclear cells of patients with depression and can be reversed by antidepressant therapy [18]. In the chronic unpredictable mild stress (CUMS)‐treated rats, NLRP3 inflammasome overactivation is observed in the liver [19, 20, 21]. In addition, the concanavalin A (ConA)‐induced murine hepatitis model exhibits aberrant NLRP3 inflammasome activation [22, 23, 24, 25], whereas relevant research in patients with AIH remains limited. Collectively, these findings demonstrate that NLRP3 activation is involved in the progression of both depression and immune‐mediated hepatitis.

Many studies have demonstrated that intestinal dysbiosis [26, 27] and intestinal barrier disruption [28, 29] contribute to the pathogenesis of AIH. Additionally, altered gut microbiota composition [30, 31, 32] and increased intestinal permeability [33] also participate in the pathogenesis of depression. Fecal microbiota transplantation (FMT) from patients with depression resulted in depressive‐like behaviors in recipient germ‐free mice [34]. Importantly, transplantation of fecal microbiota from NLRP3‐deficient mice, whose production of pro‐inflammatory cytokines was limited, alleviated depressive‐like behaviors in recipient wild‐type mice [35]. In the Mdr2^−/−^ murine model of primary sclerosing cholangitis, gut microbiota dysbiosis prompted the translocation of endotoxin to the hepatic portal system, causing severe liver injury due to pronounced NLRP3 inflammasome activation [36]. Hence, it is important to elucidate the impact of depression‐associated gut dysbiosis on the activation of the NLRP3 inflammasome during the progression of immune‐mediated hepatitis.

In this study, we investigated the effects of depression on AIH progression and elucidated the role of depression‐associated intestinal dysbiosis in this process. Specifically, we explored whether this exacerbation was mediated by the activation of the NLRP3 inflammasome in hepatic macrophages.

## 2 Materials and Methods

The Data S1 include an expanded “Supporting Informations and Methods.”

### 2.1 Design and Subjects

Consecutive patients diagnosed with AIH who visited our hepatology clinic between December 2021 and December 2023 were enrolled (*n* = 260). To eliminate the potential influence of illness duration on depressive symptoms, only patients who received standardized treatment for 2 years were recruited. AIH was diagnosed in accordance with the European Association for the Study of the Liver guidelines [37]. The inclusion criteria were as follows: (1) AIH diagnosis; (2) age > 18 years; (3) standardized treatment of prednisone and/or azathioprine for 2 years; (4) willingness to participate in the assessment of depressive symptoms performed by two trained clinicians using the 24‐item Hamilton Rating Scale for Depression (HAMD‐24); (5) absence of viral hepatitis or malignant tumors. Healthy individuals (*n* = 173) were enrolled from a health management center based on the following criteria: (1) matched for age and sex; (2) willingness to participate in the HAMD‐24 assessment; (3) no history of liver diseases; (4) no history of autoimmune diseases or malignant tumors. Informed consent was obtained from all participants. This study was approved by the Tianjin Medical University Ethics Committee, based on the ethical guidelines of the Declaration of Helsinki (revised 2013, Fortaleza, Brazil) (Ethical Approval No. IRB2021‐WZ‐191).

### 2.2 Data Collection

Two trained clinicians independently assessed depressive symptoms in patients with AIH who received standardized treatment for 2 years. During the assessment period, we systematically collected the most recent hepatic pathology reports, diagnostic imaging reports, and laboratory parameters (including white blood cells [WBC], hemoglobin [Hb], platelets [PLT], total protein [TP], albumin [ALB], globulin [GLO], total bilirubin [TB], direct bilirubin [DB], alanine aminotransferase [ALT], aspartate aminotransferase [AST], alkaline phosphatase [ALP], γ‐glutamyl transpeptidase [GGT], complement component 3 [C3], complement component 4 [C4], immunoglobulin G [IgG], IgM, and ANA). The diagnosis of cirrhosis was established based on hepatic pathology. Liver biopsy was unnecessary when clinical evidence of cirrhosis was present, including imaging report of a nodular and shrunken liver, coagulopathy, and ascites.

### 2.3 Construction of Logistic Regression Model

Comparisons of continuous variables between cirrhotic and noncirrhotic AIH patients were performed using the unpaired *t*‐test for normally distributed data and the Mann–Whitney *U* test for non‐normally distributed data. Differences in categorical variables were analyzed using the Chi‐square test. Variables showing statistically significant differences between cirrhotic and noncirrhotic AIH patients (including age, levels of ALB, GLO, ALT, AST, ALP, WBC, Hb, PLT, IgG, C3, C4, ANA titer, and comorbidities, such as depression, extrahepatic AIDs, and enlarged abdominal lymph nodes [ALN]) were further selected to build the logistic regression model. The model performance was evaluated by the calculation of area under the receiver operating characteristic curve (AUC‐ROC).

### 2.4 Animals and Treatments

Seven‐week‐old female C57BL/6 mice (18–20 g) were purchased from the Beijing Animal Study Center. The experimental animals were raised under specific‐pathogen free conditions at a constant temperature of 22°C and relative humidity of 55%. All mice received the same autoclaved water and chow under a 12‐h light/dark cycle. Female mice were selected because of the female predominance observed in patients with AIH (75%) [38] and depression (64.7%) [39]. In ConA‐induced hepatitis model, female mice exhibit more severe hepatic damage and elevated production of pro‐inflammatory cytokines than male mice [40]. An ovariectomy in female mice decreases levels of ALT and pro‐inflammatory cytokines and ameliorates ConA‐induced liver injury [40]. In contrast, orchiectomy in male mice exacerbates hepatic damage by promoting the production of pro‐inflammatory cytokines [40]. Consistent with the observations in the ConA‐induced liver injury model, female subjects are inclined to be more pro‐inflammatory and more sensitive to CUMS stimulation [41]. Furthermore, testosterone treatment correlates with improvement in depressive symptoms in men [42]. Based on these considerations, only female mice were included in the present study. Mice were randomized to different experimental groups using a random number table. After the behavioral evaluation, mice were sacrificed under anesthesia. The animal experiments complied with the ARRIVE guidelines. All experimental procedures were approved by the Ethics and Welfare Committee of Animals at Tianjin Medical University (Ethical No. IRB2023‐DWFL‐257).

### 2.5 Statistical Analysis

Statistical analysis was performed using IBM SPSS Statistics, version 26.0. The normality of the data distribution was assessed using the Kruskal–Wallis test (for a sample size ≥ 50) or Shapiro–Wilk test (for a sample size < 50). Continuous data are presented as mean ± standard deviation (SD) (normally distributed) or median (interquartile range [IQR]) (non‐normally distributed). Variables with normal distribution were analyzed using unpaired *t*‐tests or one‐way analysis of variance (ANOVA). Multiple comparisons were performed based on least significant difference (LSD) post hoc test when variances were equal and Tamhane's T2 post hoc test when variances were unequal. Nonparametric tests were conducted to compare non‐normally distributed variables. Differences in categorical variables were determined using the Chi‐square test. All reported *p*‐values were two‐sided. Statistical significance was set at *p <* 0.05. All figures were plotted using GraphPad Prism version 9.0.

## 3 Results

### 3.1 Depression Correlates With Disease Severity in Patients With AIH

Patients with AIH who received standardized treatment for 2 years (*n* = 260) were recruited. Age‐ and sex‐matched healthy participants (*n* = 173) were selected as controls (Table S1). When assessed using the HAMD questionnaire, 40.8% (106/260) of AIH patients presented with symptoms of depression, significantly higher than the rate in healthy controls (15.0%, 26/173; *p <* 0.001). Furthermore, severe symptoms of depression (HAMD score > 20) were more frequent in patients with AIH than in controls (1.2% vs. 0.0%, *p <* 0.001). AIH patients with depression presented with higher serum levels of ALT, AST, and IgG and lower serum levels of C3 and C4 than those without depression (*p =* 0.002, *p* = 0.005, *p* < 0.001, *p* = 0.044, *p* = 0.003; Table 1 and Table S2). Moreover, the comorbidities of cirrhosis, extrahepatic AIDs, and enlarged ALN were more frequent in AIH patients with depression than in those without depression (*p <* 0.001, *p <* 0.001, *p* = 0.003; Table 1 and Table S2).

**TABLE 1:** Depression correlates with disease severity in patients with autoimmune hepatitis (AIH).

	Without depression (*n* = 154)	With depression (*n* = 106)	*p*
General characteristics			
Age, median (IQR)	58.5 (48.0–65.0)	57.5 (49.0–63.0)	0.509^a^
Sex: women, *n* (%)	131 (85.1%)	94 (88.7%)	0.401^b^
Laboratory parameters (normal range)			
ALT (5.0–40.0 U/L), median (IQR)	31.0 (27.0–36.0)	36.5 (27.0–51.0)	0.002^a^
AST (8.0–40.0 U/L), median (IQR)	33.0 (28.0–38.0)	37.5 (27.0–50.0)	0.005^a^
IgG (751.0–1560.0 mg/dL), median (IQR)	1450.0 (1320.0–1530.0)	1610.0 (1400.0–1752.5)	< 0.001^a^
C3 (79.0–152.0 mg/dL), median (IQR)	100.0 (85.5–116.3)	97.8 (79.0–109.3)	0.044^a^
C4 (16.0–38.0 mg/dL), median (IQR)	21.0 (17.4–25.9)	19.5 (15.8–21.9)	0.003^a^
Disease assessment			
Cirrhosis, *n* (%)	35 (22.7%)	55 (51.9%)	< 0.001^b^
Extrahepatic AIDs, *n* (%)	47 (30.5%)	73 (68.9%)	< 0.001^b^
Enlarged ALN, *n* (%)	68 (44.2%)	67 (63.2%)	0.003^b^

*Note:* Without depression: Hamilton Depression Rating Scale (HAMD) score < 8; With depression: HAMD score ≥ 8.

Abbreviations: AIDs, autoimmune diseases; ALN, abdominal lymph node; ALT, alanine aminotransferase; AST, aspartate aminotransferase; C3, complement component 3; C4, complement component 4; Ig, immunoglobulin; IQR, interquartile range.

^a^ The statistical analysis was performed with the Mann–Whitney *U* test.

^b^ The statistical analysis was performed with the Chi‐square test.

### 3.2 Depression Promotes the Progression of Fibrosis in Immune‐Mediated Hepatitis

**FIGURE 1:**
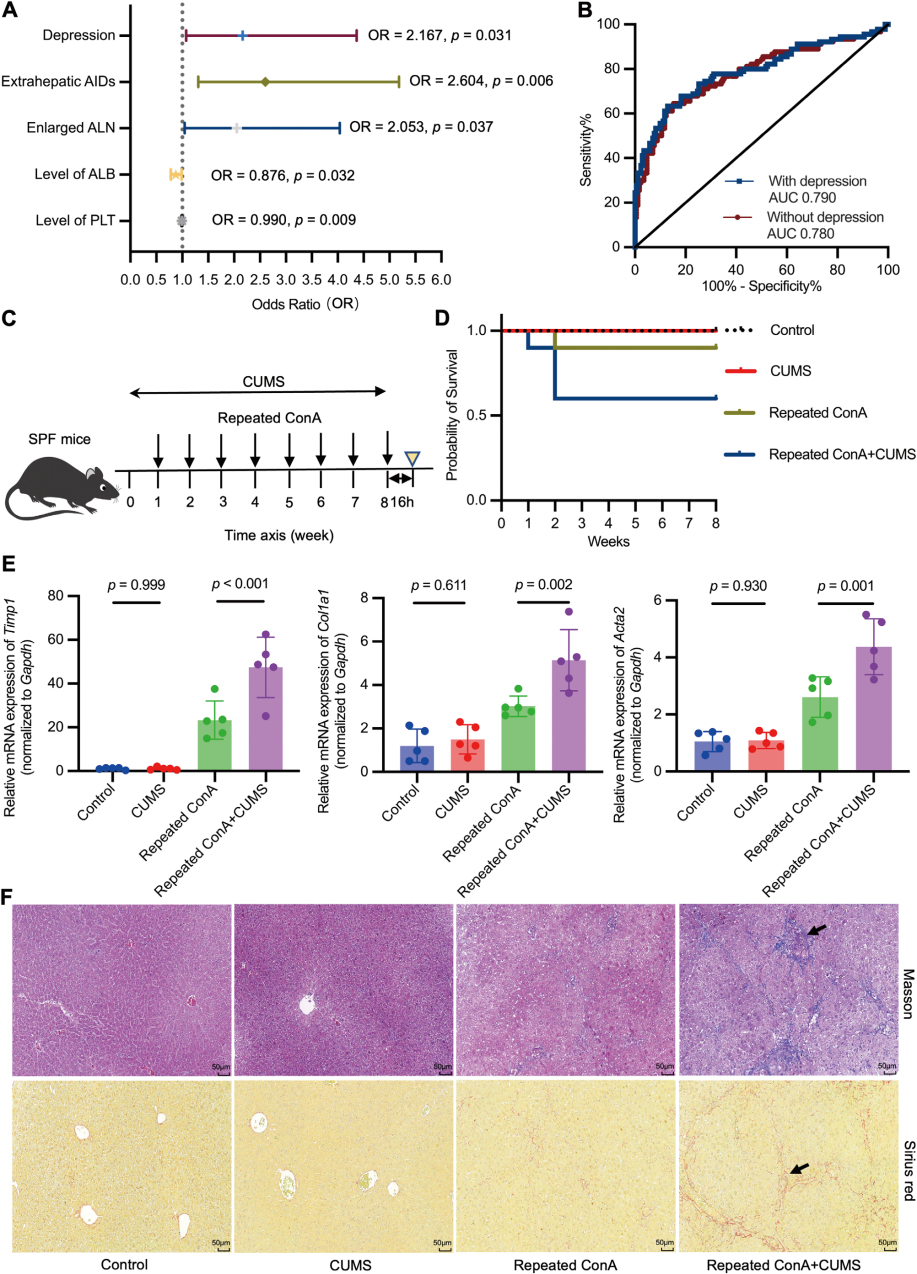
Depression promotes the progression of fibrosis in immune‐mediated hepatitis. (A) Logistic regression analysis was employed to identify indicators associated with cirrhosis in patients with autoimmune hepatitis (AIH). (B) The performance of the logistic model was evaluated by the calculation of area under the receiver operating characteristic curve (AUC‐ROC). With depression: Depression, extrahepatic autoimmune diseases (AIDs), enlarged abdominal lymph node (ALN), level of albumin (ALB), and level of platelet (PLT); Without depression: The above factors, excluding depression. (C) Study design: Mice were challenged with chronic unpredictable mild stress (CUMS) daily and concanavalin A (ConA) once a week during the course of 8 weeks. The mice were sacrificed 16 h after the last injection of ConA (yellow triangle). (D) The survival curve of different groups. (E) Quantitative real‐time polymerase chain reaction (PCR) analysis for tissue inhibitor of metalloproteinase 1 (*Timp1*), collagen, type I, alpha 1 (*Col1a1*), and Actin alpha 2 (*Acta2*) relative to glyceraldehyde‐3‐phosphate dehydrogenase (*Gapdh*) in liver tissues from the indicated groups. F = 36.411, 19.865, 29.729, all Df = 3, all *n* = 5. (F) Photo micrographs of Masson trichrome staining (upper) and Sirius red staining (lower) of the liver sections from the indicated groups. Scale bars, 50 μm. Data are presented as mean ± standard deviation (SD). Statistical comparisons between groups were performed using one‐way analysis of variance (ANOVA) and the least significant difference (LSD) post hoc test.

Among the 260 patients with AIH, 90 patients (34.6%) presented with liver cirrhosis. The rate of cirrhosis was higher in AIH patients with depression (55/106, 51.9%) than in those without depression (35/154, 22.7%) (*p* < 0.001). The following 16 parameters showed a statistical difference between AIH patients with and without cirrhosis: age, ALB, GLO, ALT, AST, ALP, WBC, Hb, PLT, IgG, C3, C4, ANA titer, and comorbidities such as depression, extrahepatic AIDs, and enlarged ALN (Table S3). In the binary logistic regression model incorporating these 16 indicators, depression (B = 0.773, odds ratio [OR] = 2.167, 95% confidence interval [CI], 1.075–4.366, *p =* 0.031) and the other four variables, including extrahepatic AIDs (B = 0.957, OR = 2.604, 95% CI: 1.308–5.185, *p =* 0.006), enlarged ALN (B = 0.719, OR = 2.053, 95% CI: 1.044–4.039, *p =* 0.037), level of ALB (B = −0.132, OR = 0.876, 95% CI: 0.777–0.988, *p =* 0.032), and level of PLT (B = −0.010, OR = 0.990, 95% CI: 0.983–0.998, *p* = 0.009), were identified as independent predictors of cirrhosis in patients with AIH (Figure 1A). The model including depression and the other four indicators exhibited a higher AUC value (0.790, 95% CI: 0.726–0.853; *p <* 0.001) compared with the model excluding the variable of depression (0.780, 95% CI: 0.716–0.844; *p <* 0.001) (Figure 1B).

To validate the impact of depression on liver fibrosis, mice with fibrosis induced by repeated ConA treatment were subjected to CUMS intervention (Figure 1C). CUMS intervention induced depressive‐like behaviors, manifested by weight loss, lower sucrose preference index, reduced total distance traveled, and decreased frequency of entering the central area (Figure S1A,D,G,J). In addition, CUMS intervention increased mortality in mice subjected to repeated ConA‐induced liver fibrosis (*p* = 0.006; Figure 1D). Importantly, the gene expression of fibrosis markers, including collagen, type I, alpha 1 (*Col1a1*), tissue inhibitor of metalloproteinase 1 (*Timp1*), and actin alpha 2 (*Acta2*), was significantly upregulated in the liver tissues of repeated ConA‐treated mice with CUMS intervention compared with those without (Figure 1E). Further, hepatic fibrosis was pathologically more severe in ConA‐treated mice with CUMS intervention than in those without, as measured by Masson trichrome staining and Sirius red staining (Figure 1F). Collectively, these observations provide experimental evidence that depression promotes fibrosis progression in immune‐mediated hepatitis.

### 3.3 Depression Promotes Hepatic NLRP3 Activation in Immune‐Mediated Hepatitis

To explore the effects of depression on the activation of NLRP3 inflammasome in patients with AIH, we collected liver tissues from AIH patients with depression, AIH patients without depression, and age‐ and sex‐matched controls (Table S4). The mRNA expression levels of *NLRP3*, *CASP1*, and *IL1B* were higher in liver biopsies from AIH patients with depression than in those without depression (Figure S2A). Immunofluorescence analysis showed a more pronounced co‐localization of NLRP3 with the macrophage marker CD68 in the hepatic tissues of depressed AIH patients compared with nondepressed patients (Figure S2B).

Next, we investigated the impact of CUMS intervention on hepatic NLRP3 expression in mice (Figure S2C). CUMS intervention resulted in elevated hepatic NLRP3 expression relative to control mice (Figure S2D,E). Furthermore, co‐localization of NLRP3 and F4/80 was more frequent in hepatic sections of CUMS‐treated mice compared with control mice (Figure S3A). Previous studies have demonstrated that CD11b^+^Ly6C^hi^ macrophages, a subset of pro‐inflammatory cells, released higher amounts of NLRP3 and IL1B than CD11b^+^Ly6C^lo^ and CD11b^−^Ly6C^−^ cells [43]. This prompted us to investigate the infiltration of CD45^+^CD11b^+^Ly6C^hi^ macrophages in mice. Notably, CD45^+^CD11b^+^Ly6C^hi^ cells accumulated to a greater extent in the liver and spleen of CUMS‐treated mice than in control mice (Figures S2F and S3B).

MCC950, a highly specific NLRP3 inhibitor, was used to determine whether targeting NLRP3 could alleviate liver injury (Figure S2C). After ConA‐treated mice were subjected to CUMS intervention, serum ALT and AST levels and hepatic NLRP3 expression were significantly elevated, which could be alleviated by MCC950 treatment (Figures S2D,E and S3A,C). Significantly, histological analysis using hematoxylin and eosin (H&E) staining revealed severe portal inflammatory cell infiltration in the liver of ConA mice with CUMS intervention, which could be ameliorated by MCC950 (Figure S3D). Additionally, MCC950 treatment reduced the accumulation of CD45^+^CD11b^+^Ly6C^hi^ macrophages in the liver and spleen of ConA‐treated mice subjected to CUMS intervention (Figures S2F and S3B).

### 3.4 Depression Exacerbates the Disruption of the Intestinal Barrier Function in Immune‐Mediated Hepatitis

**FIGURE 2:**
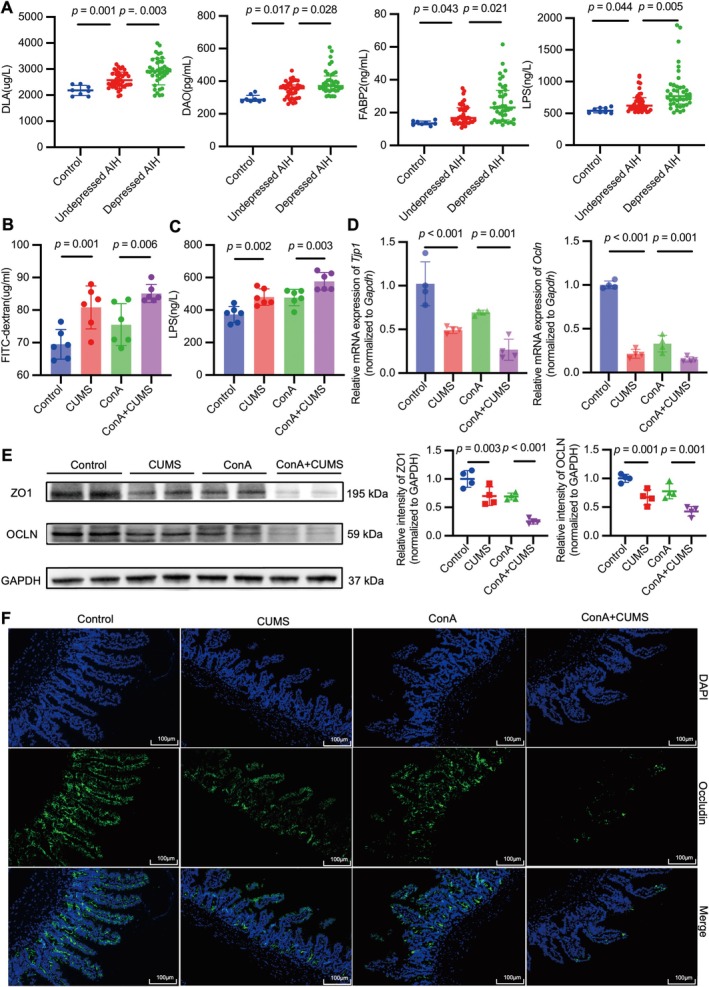
Depression exacerbates the disruption of the intestinal barrier function in immune‐mediated hepatitis. (A) Serum levels of D‐lactic acid (DLA), diamine oxidase (DAO), fatty acid‐binding protein‐2 (FABP2), and lipopolysaccharide (LPS) were analyzed by enzyme‐linked immunosorbent assay (ELISA) in the control group (*n* = 8), AIH without depression group (*n* = 40), and AIH with depression group (*n* = 40). *F* = 13.430, KW = 20.266, 18.607, 22.166, all DF = 2. (B, C) The serum concentration of fluorescein isothiocyanate‐dextran (FITC‐D) in vivo permeability assay (B) and the serum concentration of LPS by ELISA (C). *F* = 9.639, 15.902, both DF = 3, both *n* = 6. (D) PCR analysis of tight junction protein 1 (*Tjp1*) or occludin (*Ocln*) relative to *Gapdh* in the intestine from the indicated groups. *F* = 20.463, 173.780, both DF = 3, both *n* = 4. (E) Western blot analysis of Zonula occludens protein 1 (ZO1) or OCLN relative to GAPDH in the intestine from the indicated groups. *F* = 28.575, 19.922, both DF = 3, both *n* = 4. (F) Representative immunofluorescence staining with occludin in the intestine from the indicated groups. Scale bars, 100 μm. The data for B–E are presented as mean ± SD. Statistical comparisons between groups were performed using one‐way ANOVA and the LSD post hoc test. The data for DLA are expressed as mean ± SD (analyzed by one‐way ANOVA with Tamhane's T2 post hoc test), whereas the data for DAO, FABP2, and LPS are presented as median (interquartile range [IQR]) (analyzed by nonparametric tests).

To investigate the role of depression in intestinal barrier dysfunction in patients with AIH, we measured serum biomarkers of intestinal permeability, including D‐lactic acid (DLA), diamine oxidase (DAO), fatty acid‐binding protein‐2 (FABP2), and lipopolysaccharide (LPS). Intestinal barrier dysfunction was more common in AIH patients with depression than in those without depression (Figure 2A).

Compared with vehicle control mice, exposure to CUMS increased intestinal permeability, as reflected by increased levels of fluorescein isothiocyanate‐dextran (FITC‐D) and LPS in the serum and decreased levels of Zonula occludens protein 1 (ZO1) and occludin (OCLN) in the intestine (Figure 2B–E). Immunofluorescence staining of occludin further confirmed more severe intestinal barrier disruption in CUMS‐treated mice (Figure 2F). In particular, compared with mice treated with ConA alone, those with combined exposure to CUMS and ConA intervention exhibited more severe intestinal barrier disruption, as indicated by altered levels of FITC‐D, LPS, ZO1, and OCLN (Figure 2B–F). Overall, depression can exacerbate the disruption of the intestinal barrier function in immune‐mediated hepatitis.

### 3.5 Mirtazapine Exerts Protective Effects by Alleviating the Disruption of the Intestinal Barrier and the Overactivation of NLRP3 in Mice

**FIGURE 3:**
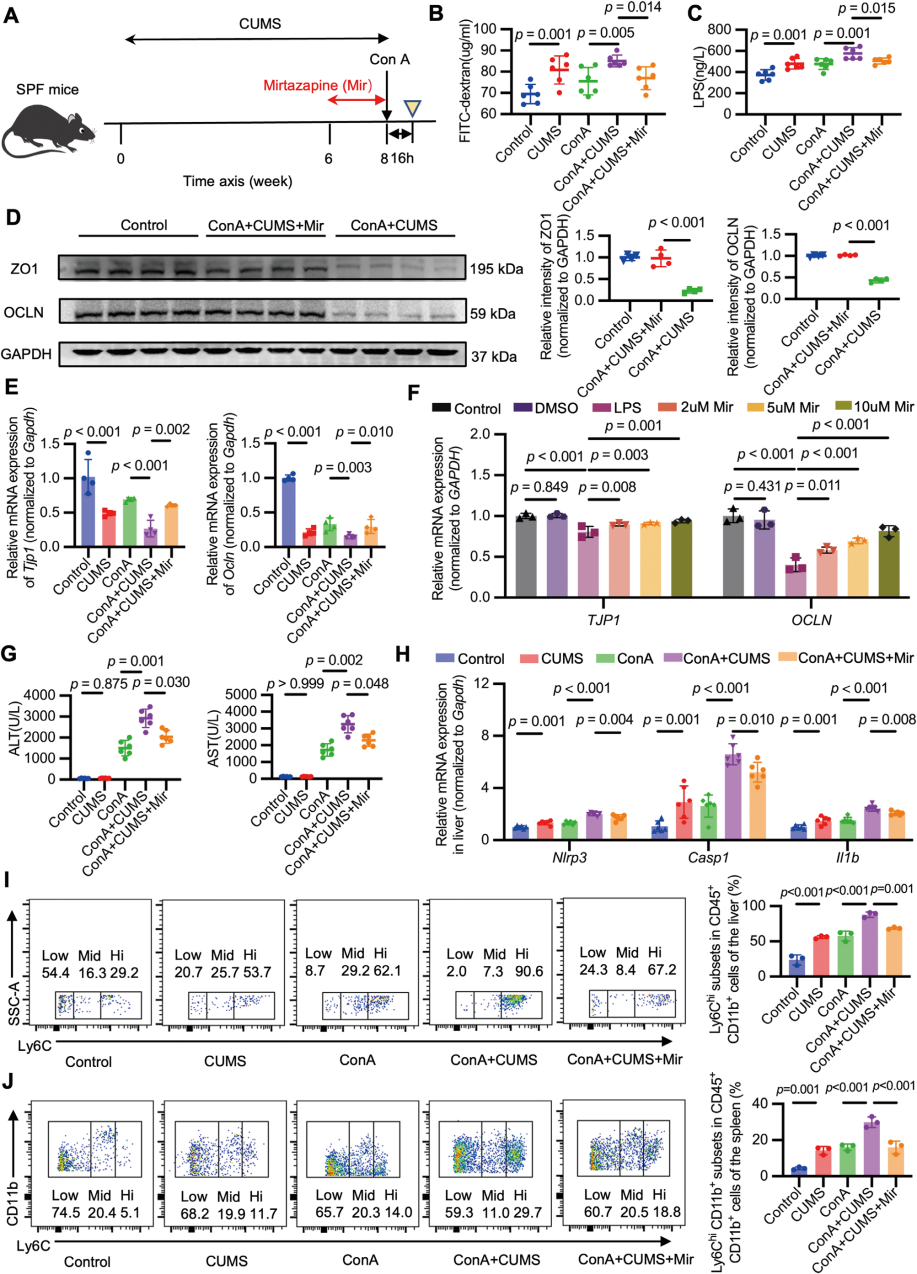
Mirtazapine exerts protective effects by alleviating the disruption of the intestinal barrier and the overactivation of NLR family pyrin domain containing 3 (NLRP3) inflammasome in mice. (A) Study design: CUMS‐induced depression mice were intraperitoneally injected with mirtazapine daily during the last 2 weeks. Two hours after the last injection of mirtazapine, mice were intravenously injected with ConA. The mice were sacrificed 16 h after ConA injection (yellow triangle). (B, C) Serum concentration of FITC‐D in vivo permeability assay (B) and the serum concentration of LPS by ELISA (C). (B) *F* = 7.216, DF = 4, *n* = 6. (C) *F* = 14.250, DF = 4, *n* = 6. (D) Western blots analysis of ZO1 or OCLN relative to GAPDH in the intestine from the indicated groups. *F* = 51.304, 328.339, both DF = 2, both *n* = 4. (E) PCR analysis of *Tjp1* or *Ocln* relative to *Gapdh* in the intestine from the indicated groups. *F* = 19.112, 96.428, both DF = 4, both *n* = 4. (F) PCR analysis of *TJP1* or *OCLN* relative to *GAPDH* in LPS‐treated Caco‐2 monolayers. *F* = 14.148, 28.119, both DF = 5, both *n* = 3. (G) The serum levels of alanine aminotransferase (ALT) and aspartate aminotransferase (AST) in mice from the indicated groups. *F* = 110.511, 105.418, both DF = 4, both *n* = 6. (H) PCR analysis of *Nlrp3*, caspase‐1 *(Casp1)*, and interleukin 1 beta (*Il1b*) relative to *Gapdh* in liver samples from the indicated groups. *F* = 39.049, 39.358, 36.442, all DF = 4, all *n* = 6. (I, J) The proportions of CD45^+^CD11b^+^Ly6C^hi^ subsets among CD45^+^CD11b^+^ cells from the liver (I) and spleen (J) were quantified using flow cytometry analysis. (I) *F* = 67.774, DF = 4, *n* = 3. (J) *F* = 42.182, DF = 4, *n* = 3. All the values are shown as mean ± SD. Statistical significance was calculated using one‐way ANOVA with LSD post hoc test (B–F, H–J) and Tamhane's T2 post hoc test (G).

The antidepressant mirtazapine was administered to investigate its effect on the destruction of the intestinal barrier integrity (Figure 3A). Mirtazapine alleviated intestinal permeability in ConA mice with CUMS intervention, as demonstrated by decreased levels of FITC‐D and LPS in the serum and increased levels of ZO1 and OCLN in the intestine (Figure 3B–E). Immunofluorescence staining of occludin further confirmed the protective role of mirtazapine in maintaining intestinal epithelial integrity (Figure S5A). As it has been demonstrated that LPS could cause intestinal barrier dysfunction in Caco‐2 epithelial cell monolayers [44, 45, 46], we next explored whether mirtazapine exerted a protective effect on LPS‐treated Caco‐2 cells in vitro. The results supported our hypothesis and demonstrated that mirtazapine increased the mRNA expression of *TJP1* and *OCLN* in a concentration‐dependent manner, which further proved its protective effect on the intestinal barrier function (Figure 3F).

Next, we investigated whether mirtazapine participated in immune regulation. In ConA mice with CUMS intervention, mirtazapine reduced the levels of ALT and AST in the serum (Figure 3G) and the expression of NLRP3 in the liver (Figure S2E, Figure 3H, and Figure S5B). Additionally, flow cytometry analysis showed that CD45^+^CD11b^+^Ly6C^hi^ cells accumulated to a lower extent in the liver and spleen after mirtazapine administration (Figure 3I,J). Moreover, in the isolated murine peritoneal macrophages, LPS stimulation upregulated the expression of *Nlrp3*, *Casp1*, and *Il1b*, which was counteracted by mirtazapine pretreatment in a concentration‐dependent manner (Figure S5C). These research findings demonstrate that mirtazapine exerts protective effects by alleviating the disruption of the intestinal barrier function and the overactivation of the NLRP3 inflammasome.

### 3.6 Colonization With Intestinal Microbiota of Patients With MDD Disrupts Intestinal Epithelial Integrity and Overactivates Hepatic NLRP3 Inflammasome in Mice

**FIGURE 4:**
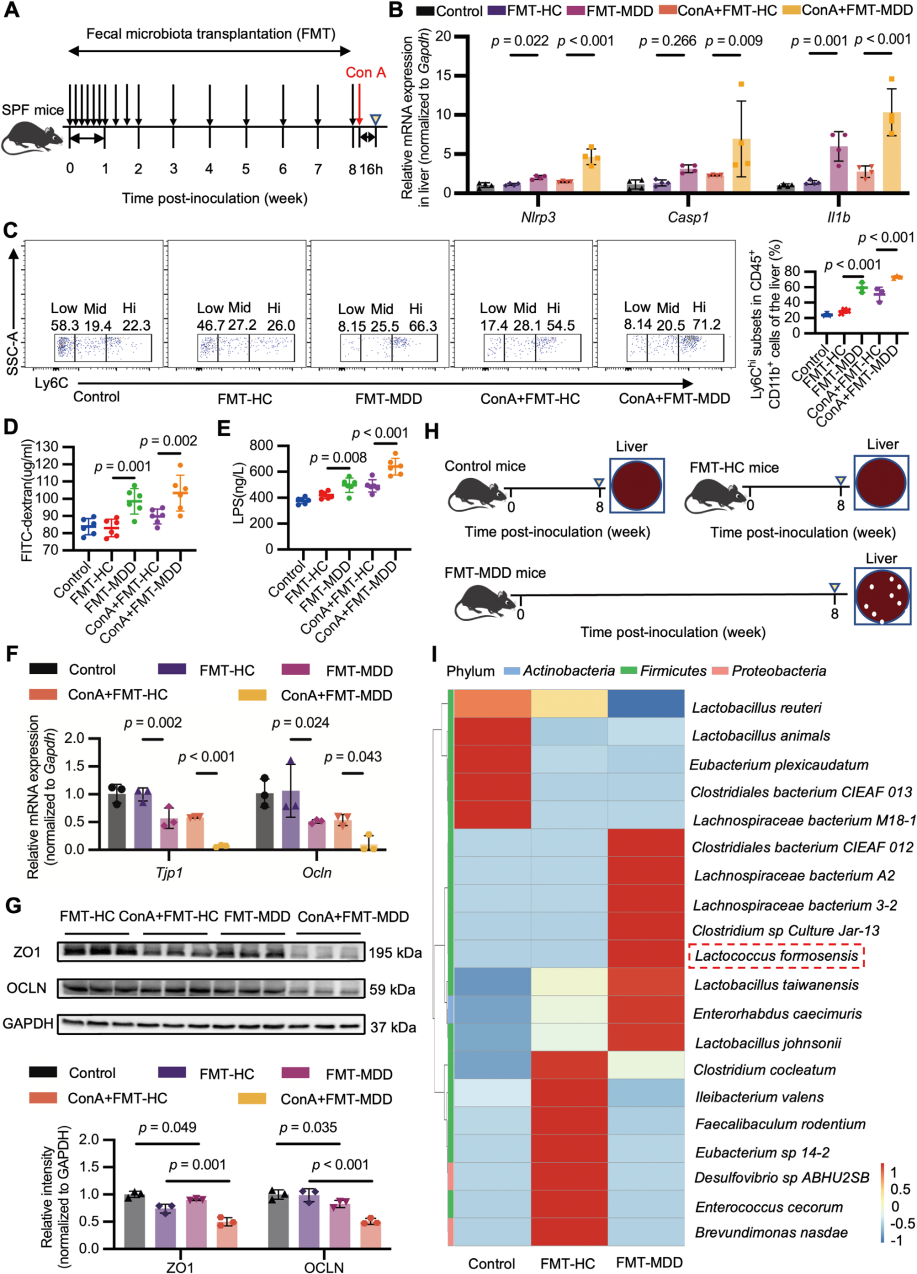
Colonization with intestinal microbiota of patients with major depressive disorder (MDD) disrupts intestinal epithelial integrity and overactivates hepatic NLRP3 inflammasome in mice. (A) Study design: Seventy‐two hours after antibiotic discontinuation, mice received fecal microbiota transplantation (FMT) from patients with MDD or healthy controls (HC). The mice were sacrificed 16 h after ConA injection (yellow triangle). (B) PCR analysis of *Nlrp3*, *Casp1*, and *Il1b* relative to *Gapdh* in liver biopsies from the indicated groups. *F* = 37.843, 4.630, 23.158, all DF = 4, all *n* = 4. (C) The proportions of CD45^+^CD11b^+^Ly6C^hi^ cells among CD45^+^CD11b^+^ cells in the liver were quantified by flow cytometry analysis. F = 46.149, DF = 4, *n* = 3. (D, E) The serum concentration of FITC‐D in vivo permeability assay (D) and the serum concentration of LPS by ELISA (E). (D) *F* = 10.499, DF = 4, *n* = 6. (E) *F* = 27.756, DF = 4, *n* = 6. (F) PCR analysis of *Tjp1* or *Ocln* relative to *Gapdh* in the intestine from the indicated groups. *F* = 29.744, 7.447, both DF = 4, both *n* = 3. (G) Western blot analysis of ZO1 or OCLN relative to GAPDH in the intestine from the indicated groups. *F* = 38.268, 21.866, both DF = 3, both *n* = 3. (H) Gut‐derived *Lactococcus formosensis* was successfully isolated from the liver of FMT‐MDD mice. (I) 16S rRNA gene sequencing of intestinal microbiota from the indicated groups. All the values are presented as mean ± SD. Statistical difference was calculated by one‐way ANOVA with LSD post hoc test.

Colonization with MDD microbiota resulted in depressive‐like behaviors in mice, manifested by weight loss, lower sucrose preference index, reduced total distance traveled, and decreased frequency of entering the central area, in contrast to those with control microbiota (Figure 4A and Figure S1B,E,H,K). Furthermore, when subjected to ConA injection, mice receiving gut microbiota transplantation from patients with MDD exhibited more severe liver damage than those harboring control microbiota (Figure S4A), as evidenced by elevated serum levels of ALT and AST (Figure S4B) and hepatic expression of NLRP3 (Figure 4B and Figure S4C). The co‐localization of F4/80 and NLRP3 further indicated the involvement of hepatic macrophages in liver injury (Figure S4D). Additionally, CD45^+^CD11b^+^Ly6C^hi^ cells accumulated to a greater extent in the liver and spleen of FMT‐MDD and ConA + FMT‐MDD mice compared with FMT‐HC and ConA + FMT‐HC mice (Figure 4C and Figure S4E).

Next, we demonstrated that transplantation of MDD microbiota notably increased intestinal permeability and further aggravated endotoxemia (Figure 4D,E). Mice colonized with MDD microbiota exhibited decreased levels of ZO1 and OCLN in the intestine compared with those colonized with control microbiota (Figure 4F,G). To identify the presence of bacteria in the extraintestinal organs, the liver, spleen, and mesenteric lymph nodes (MLN) from mice colonized with microbiota from patients with MDD (*n* = 4) were aseptically collected and cultured. Of these, bacterial clones were isolated from the livers of 3 out of 4 mice when cultured on Columbia blood agar plates under aerobic or anaerobic conditions. However, no bacterial clones could be grown from the spleen or MLN of mice receiving FMT from MDD patients. Similarly, no bacterial growth was observed in any organs from the control mice or mice receiving FMT from healthy controls. The bacteria were subsequently identified as *Lactococcus formosensis* using 16S rRNA gene sequencing (Figure 4H and Figure S6D). Simultaneously, we confirmed that the abundance of *L. formosensis* was higher in stool samples of mice receiving FMT from patients with MDD than in those receiving FMT from healthy controls (Figure 4I). Hence, these data collectively demonstrate that colonization with MDD microbiota can disrupt intestinal epithelial integrity and promote the activation of hepatic NLRP3 inflammasome in mice.

### 3.7 Translocation of Gut‐Derived *L. formosensis* Overactivates the Hepatic NLRP3 Inflammasome and Induces Liver Injury in Mice

**FIGURE 5:**
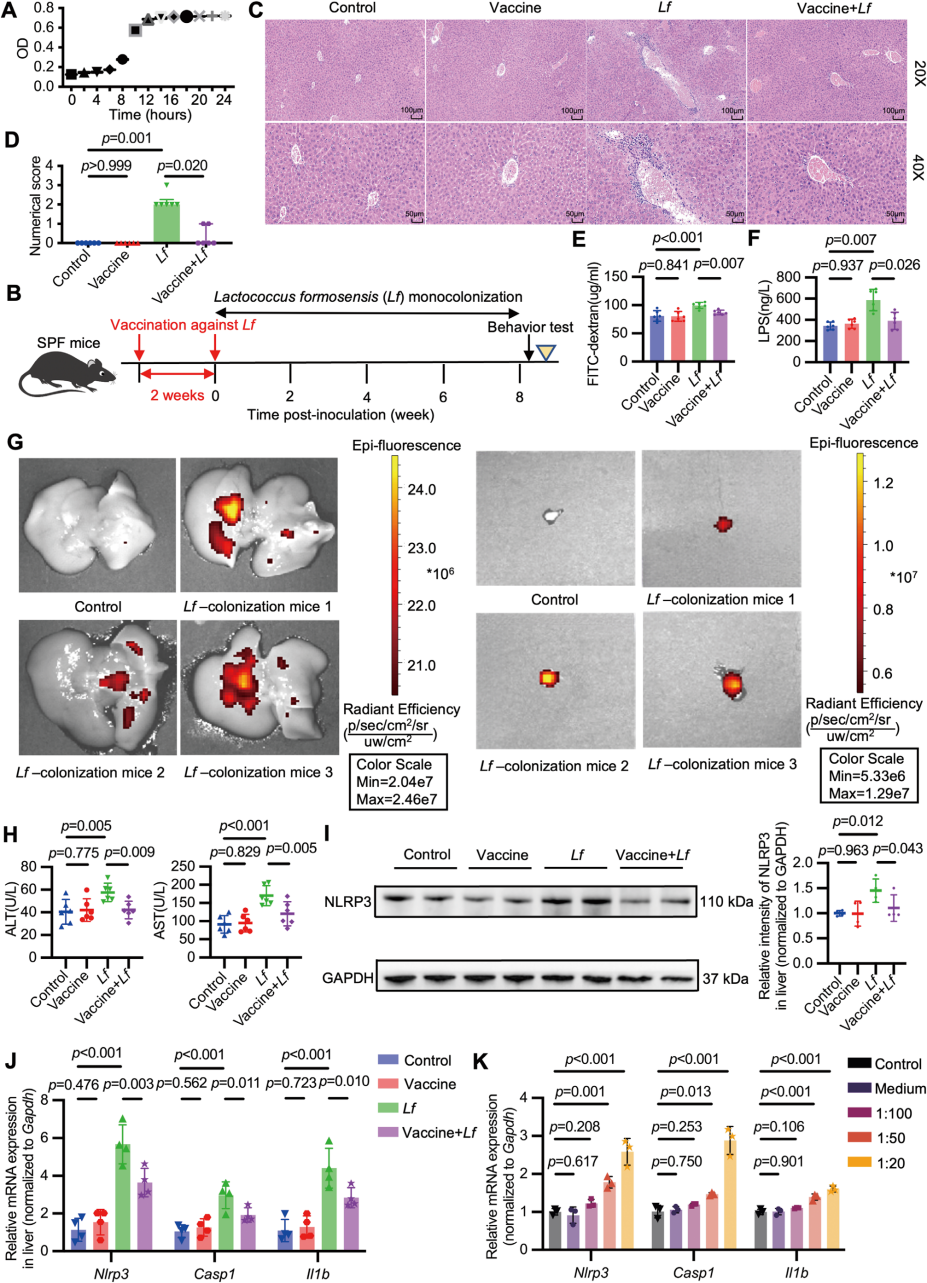
Translocation of gut‐derived *L. formosensis* overactivates the hepatic NLRP3 inflammasome and induces liver injury in mice. (A) The growth curve of *L. formosensis* was determined by optical density (OD) measurements at 600 nm. (B) Study design: Seventy‐two hours after antibiotic discontinuation, mice were administered *L. formosensis* once a week during the course of 8 weeks by oral gavage. Before the first gavage, the vaccine targeting *L. formosensis* ‐specific DNA was intramuscularly injected once a week during the course of 2 weeks. The evaluation of depressive‐like behaviors was conducted 24 h after the last administration. The yellow triangle represents the point of analysis. (C) Representative hematoxylin and eosin (H&E) staining images of liver tissues were shown (Scale bars: 100 μm, 50 μm). (D) The severity of inflammatory cell infiltration was graded using the 4‐point scoring system. KW = 19.677, DF = 3, *n* = 6. (E, F) The concentration of FITC‐D in vivo permeability assay (E) and the serum concentration of LPS by ELISA (F). (E) *F* = 9.190, DF = 3, *n* = 6. (F) *F* = 15.299, DF = 3, *n* = 6. (G) The translocation of green fluorescent protein (GFP)‐labeled *L. formosensis* to liver (left) or mesenteric lymph nodes (MLN) (right). Fluorescence in the liver or MLN indicated bacterial translocation. (H) The serum levels of ALT and AST in mice from the indicated groups. *F* = 4.516, 10.612, both DF = 3, both *n* = 6. (I) Western blot analysis of NLRP3 relative to GAPDH in the liver from the indicated groups. *F* = 3.984, DF = 3, *n* = 4. (J) PCR analysis of *Nlrp3*, *Casp1*, and *Il1b* relative to *Gapdh* in the liver from the indicated groups. *F* = 28.603, 12.039, 18.164, all DF = 3, all *n* = 4. (K) PCR analysis of *Nlrp3*, *Casp1*, and *Il1b* relative to *Gapdh* in the isolated murine peritoneal macrophages treated with the supernatant of *L. formosensis* in a concentration‐dependent manner (1:100, 1:50, 1:25). *F* = 34.235, 52.209, 42.390, all DF = 4, all *n* = 3. The data for E–F, H–K are presented as mean ± SD. Statistical significance was calculated by one‐way ANOVA with LSD post hoc test (E, H–K) and Tamhane's T2 post hoc test (F). The data for D are presented as median (IQR) and were analyzed using nonparametric tests.

The growth and genetic characteristics of *L. formosensis* were summarized in Figure 5A and Figure S6A–D. We found that mice monocolonized with *L. formosensis* exhibited depressive‐like behaviors (e.g., weight loss, lower sucrose preference index, reduced total distance traveled, and decreased frequency of entering the central area) (Figure S1C,F,I,L). Histological analysis using H&E staining revealed severe portal inflammatory cell infiltration in the liver of mice monocolonized with *L. formosensis* , which was ameliorated by a specific vaccine targeting *L. formosensis* (Figure 5B–D). In addition, mice monocolonized with *L. formosensis* exhibited disrupted intestinal epithelial integrity, manifested by elevated serum levels of FITC‐D and LPS. Notably, the elevated levels of FITC‐D and LPS were counteracted by vaccination (Figure 5E,F). Consistently, mice monocolonized with *L. formosensis* showed bacterial translocation from the intestine to the liver and MLN (Figure S6D), as confirmed by green fluorescent protein (GFP)‐labeled bacteria (Figure 5G). Vaccination prevented bacterial translocation, as no growth of *L. formosensis* was observed in any organ of *L. formosensis* monocolonized mice pretreated with the vaccine.

Monocolonization with *L. formosensis* elevated serum levels of ALT and AST (Figure 5H) and hepatic expression of NLRP3 (Figure 5I,J, Figure S6E), which could also be alleviated by the specific vaccine against *L. formosensis* . In the isolated murine peritoneal macrophages, the supernatant of *L. formosensis* upregulated the expression of *Nlrp3*, *Casp1*, and *Il1b* in a concentration‐dependent manner (Figure 5K). In conclusion, monocolonization with *L. formosensis* is sufficient to disrupt intestinal epithelial integrity and lead to subsequent bacterial translocation to the liver, which can amplify NLRP3‐mediated liver injury.

## 4 Discussion

This study demonstrated that depression exacerbated immune‐mediated hepatitis through the gut‐liver axis. Clinically, our findings highlighted a high prevalence of depression in patients with AIH and further demonstrated its significant association with an increased risk of cirrhosis and intestinal barrier disruption. Mechanistically, we revealed that the detrimental effects of depression were mediated by intestinal barrier dysfunction and NLRP3 overactivation. The translocation of gut‐derived *L. formosensis* to the liver played pivotal roles in the overactivation of the hepatic NLRP3 inflammasome and consequent liver injury.

Previous studies have demonstrated a high prevalence of depression in patients with AIH [2, 47]. Depression, a key factor responsible for nonadherence to standard treatment, increases the risk of relapse in patients with AIH [5]. Moreover, depression is related to adverse clinical outcomes in patients with chronic liver disease [48] and is recognized as an important risk factor for liver fibrosis [6, 7, 8]. Consistent with previous studies, we observed that depression was common in patients with AIH (40.8%) and correlated with disease severity. Additionally, depression was an independent risk factor for cirrhosis in patients with AIH. We validated the above clinical results in a mouse model. Repeated ConA injections increased susceptibility to liver fibrosis in mice [49]. Based on this liver fibrosis model, we demonstrated that CUMS intervention promoted fibrosis progression in mice repeatedly injected with ConA. These results highlight the clinical significance of identifying and managing depression in AIH.

Patients with depression commonly exhibit systemic inflammation with elevated levels of pro‐inflammatory cytokines in plasma [50]. The NLRP3 inflammasome, involved in CASP1 activation, exerts a crucial effect on depression [15, 16, 17]. In the livers of ConA‐induced hepatitis mice, the expression levels of NLRP3, CASP1, and IL1B are significantly upregulated [25]. The NLRP3 inflammasome activation also participates in the liver inflammation of other forms of immune‐mediated hepatitis [51, 52]. Consistent with previous findings, we demonstrated upregulated expression of *Nlrp3*, *Casp1*, and *Il1b* in the livers of CUMS‐treated mice. Furthermore, CUMS intervention upregulated the expression of hepatic *Nlrp3*, *Casp1*, and *Il1b* in ConA‐treated mice, which was attenuated by the highly specific NLRP3 inhibitor MCC950. In patients with AIH, elevated expression levels of *NLRP3*, *CASP1*, and *IL1B* were more commonly detected in the liver biopsies from depressed AIH patients compared with nondepressed patients. Taken together, these data imply that depression may promote the activation of NLRP3 and subsequent disease progression in immune‐mediated hepatitis.

Gut‐derived systemic inflammation acts as a driver of depression in chronic liver disease [53]. Gut dysbiosis has been identified as a shared pathogenic mechanism in AIH [26, 27] and depression [30, 31, 32]. A recent study suggested that gut‐derived *Enterococcus gallinarum* could translocate to the liver through the disrupted intestinal barrier, inducing autoimmunity in patients and mouse models of AIH [54]. Another study demonstrated that gut‐derived *Klebsiella pneumonia* promoted intestinal barrier dysfunction through epithelial pore formation, leading to bacterial translocation and subsequent hepatic inflammation in primary sclerosing cholangitis [55]. In depression, gut dysbiosis also causes impairment of intestinal mucosal permeability and culminates in increased bacterial translocation from the intestine to extraintestinal organs [33, 56]. In this study, we isolated *L. formosensis* from the livers of mice colonized with the microbiota of patients with MDD. Furthermore, disruption of the intestinal barrier integrity and overactivation of the hepatic NLRP3 were observed in mice receiving FMT from patients with MDD and in mice monocolonized with *L. formosensis* . Therefore, our data provide evidence for a mechanism through which depression aggravates immune‐mediated hepatitis, which involves hepatic NLRP3 inflammasome overactivation induced by depression‐associated intestinal dysbiosis.

In our study, mirtazapine, a competitive inhibitor of serotonin type 2 and type 3 receptors, was used to investigate its effect on immune‐mediated hepatitis. Patients with depression are more likely to develop ulcerative colitis. Interestingly, mirtazapine protects against ulcerative colitis by alleviating behavioral abnormalities and modulating neuronal excitability in the brain [57]. In primary biliary cholangitis, mirtazapine protects against a poor prognosis by decreasing the incidence of cirrhosis and mortality [58]. In mouse models, mirtazapine attenuates ConA‐induced hepatic injury in a concentration‐dependent manner [59]. In this study, we verified that mirtazapine conferred protective effects by alleviating the impairment of intestinal barrier integrity and abnormal activation of the NLRP3 inflammasome in ConA‐treated mice with CUMS intervention. Therefore, investigating the therapeutic potential of mirtazapine in patients with AIH, particularly those with comorbid depression, is of vital clinical importance.

This study presented several key innovations. Clinically, it highlighted the necessity of considering depressive symptoms in patients with AIH, given their association with an increased risk of cirrhosis and disease progression. Mechanistically, we verified that depression exacerbated immune‐mediated hepatitis by impairing the intestinal barrier and overactivating the hepatic NLRP3 inflammasome. Critically, gut dysbiosis played a pivotal role in depression‐related liver injury. Our study is the first to successfully isolate gut‐derived *L. formosensis* from the livers of mice receiving FMT from patients with MDD, and to elucidate the pivotal role of *L. formosensis* in immune regulation. Furthermore, a vaccine targeting *L. formosensis* was shown to prevent bacterial translocation and alleviate liver injury in mice, demonstrating therapeutic potential. These findings pave the way for considering mirtazapine as a promising therapeutic option for AIH.

However, several limitations of this study must be acknowledged. First, it was a single‐center investigation, which necessitates further validation in multicenter cohorts. Second, the lack of data on the clinical efficacy of mirtazapine limits the clinical applicability of our findings. Additionally, the clinical relevance of *L. formosensis* translocation, including its prevalence in human patients and its contribution to disease progression, remains to be fully established. Finally, it was difficult to determine causality between depression and the observed results. From the perspective of neuroimmune circuits, the frequent comorbidity of depression in patients with AIH may be ascribed to the interaction between the brain and the immune system. Therefore, future studies should explore the interaction between depression and AIH from different perspectives.

## 5 Conclusions

**FIGURE 6:**
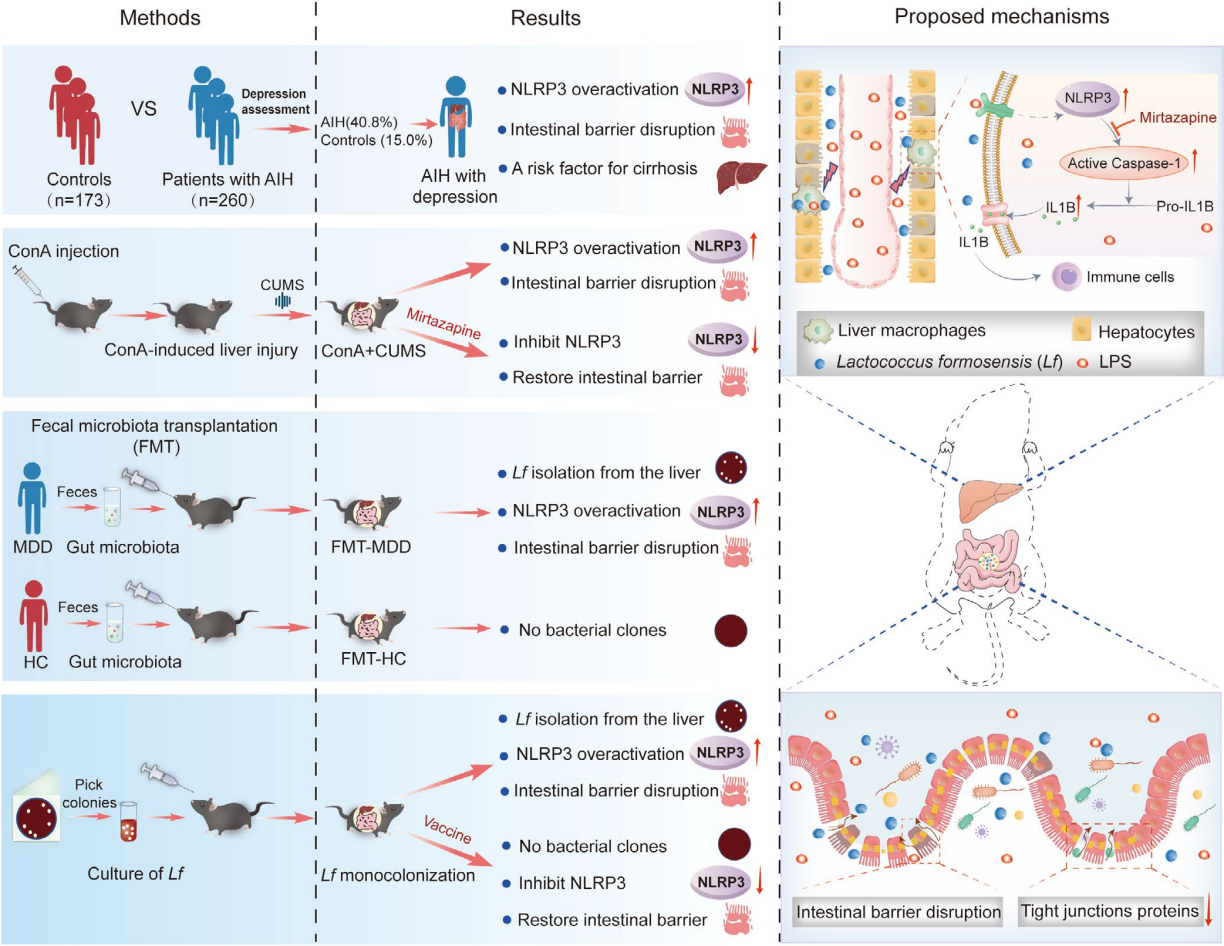
Illustration depicting the role of depression and depression‐associated gut microbiota in immune‐mediated hepatitis. Depression is common in patients with AIH and is a risk factor for cirrhosis. AIH patients with depression, mice colonized with microbiota from patients with MDD, and ConA‐treated mice with CUMS intervention exhibit intestinal barrier dysfunction and hepatic NLRP3 inflammasome overactivation. Gut‐derived *L. formosensis* , isolated from the livers of mice colonized with microbiota from patients with MDD, plays pivotal roles in the disruption of intestinal epithelial integrity and overactivation of hepatic NLRP3 inflammasome. Antidepressants mirtazapine and vaccines against *L. formosensis* can alleviate liver injury in mice.

In conclusion, depression, a common mental disorder in patients with AIH, contributed to the progression of fibrosis. As graphically summarized (Figure 6), liver inflammation driven by depression was associated with intestinal barrier dysfunction and NLRP3 overactivation in liver macrophages. *L. formosensis* , isolated from the livers of mice colonized with microbiota from patients with depression, played pivotal roles in the disruption of intestinal epithelial integrity and overactivation of NLRP3 in the liver. Antidepressants and vaccines against *L. formosensis* may be strategies for preventing and treating patients with AIH, particularly those with depression.

## Author Contributions

**Simin Zhou:** conceptualization, data curation, formal analysis, writing – original draft. **Liping Guo:** data curation, investigation, visualization. **Nian Chen:** formal analysis, software, visualization. **Haifeng Liu:** data curation, writing – review and editing. **Xin Liu:** conceptualization, writing – original draft. **Jiwen Li:** conceptualization. **Shijing Dong:** methodology. **Jiangpeng Liu:** validation. **Xiaoyi Wang:** methodology. **Ying Ran:** methodology. **Man Liu:** conceptualization. **Hongyu Chu:** data curation. **Yanni Li:** methodology. **Hui Yang:** validation. **Jingwen Zhao:** conceptualization, project administration, supervision, visualization. **Lu Zhou:** funding acquisition, resources, writing – original draft.

## Funding

This work was supported by National Natural Science Foundation of China (81860109) and Natural Science Foundation of Tianjin Municipality (21JCZDJC00880).

## Disclosure

The authors have nothing to report.

## Ethics Statement

This study involving human participants was approved by the Tianjin Medical University Ethics Committee, based on the ethical guidelines of the Declaration of Helsinki (revised 2013, Fortaleza, Brazil) (Ethical Approval No. IRB2021‐WZ‐191). The animal experiment was approved by the Ethics and Welfare Committee of Animals at Tianjin Medical University (Ethical No. IRB2023‐DWFL‐257).

## Consent

Informed consent was obtained from all participants.

## Conflicts of Interest

The authors declare no conflicts of interest.

## Supporting information


**Data S1:** The Data S1 includes Supporting Informations and Methods, Supplementary Figures, and Supplementary Tables.
**Figure S1:** Assessment of depressive‐like behaviors in mice.
**Figure S2:** Depression promotes the activation of NLRP3 in immune‐mediated hepatitis.
**Figure S3:** A highly specific NLRP3 inhibitor MCC950 exerts protective effects in mice.
**Figure S4:** Fecal microbiota transplantation from patients with MDD induces adverse effects in recipient mice.
**Figure S5:** Mirtazapine exerts protective effects by alleviating the disruption of intestinal barrier and the overactivation of NLRP3 in mice.
**Figure S6:** Translocation of gut‐derived *L. formosensis* drives hepatic NLRP3 overactivation and liver injury in mice.
**Table S1:** Comparison of general characteristics and laboratory parameters between patients with AIH and healthy controls.
**Table S2:** Comparison of general characteristics and laboratory parameters between AIH patients with and without depression.
**Table S3:** Comparison of general characteristics and laboratory parameters between AIH patients with and without cirrhosis.
**Table S4:** Clinical characteristics of participants included in the PCR‐based analysis of hepatic NLRP3 pathway expression.

## Data Availability

Data are available on request from the corresponding authors.

## References

[1] C. D. Slooter , F. F. van den Brand , A. Lleo , et al., “Lack of Complete Biochemical Response in Autoimmune Hepatitis Leads to Adverse Outcome: First Report of the IAIHG Retrospective Registry,” Hepatology 79, no. 3 (2024): 538–550. 37676683 10.1097/HEP.0000000000000589

[2] C. Schramm , I. Wahl , C. Weiler‐Normann , et al., “Health‐Related Quality of Life, Depression, and Anxiety in Patients With Autoimmune Hepatitis,” Journal of Hepatology 60, no. 3 (2014): 618–624. 24240053 10.1016/j.jhep.2013.10.035

[3] L. Yang , B. Gu , X. Wang , Q. Ren , M. Shen , and D. Su , “Association of Disease Activity With Depression and Anxiety in Systemic Lupus Erythematosus: A Comparison of SLEDAI‐2K and SLE‐DAS,” Rheumatology (Oxford) 64, no. 2 (2025): 632–638. 38305645 10.1093/rheumatology/keae070

[4] Y. Qian , Y. Chen , L. Liu , T. Wu , X. Chen , and G. Ma , “Depression and Anxiety in Inflammatory Bowel Disease: Mechanisms and Emerging Therapeutics Targeting the Microbiota‐Gut‐Brain Axis,” Frontiers in Immunology 16 (2025): 1676160. 41280932 10.3389/fimmu.2025.1676160 PMC12634526

[5] S. Sockalingam , D. Blank , N. Abdelhamid , S. E. Abbey , and G. M. Hirschfield , “Identifying Opportunities to Improve Management of Autoimmune Hepatitis: Evaluation of Drug Adherence and Psychosocial Factors,” Journal of Hepatology 57, no. 6 (2012): 1299–1304. 22871503 10.1016/j.jhep.2012.07.032

[6] D. Kim , B. B. Dennis , G. Cholankeril , and A. Ahmed , “Association Between Depression and Metabolic Dysfunction‐Associated Fatty Liver Disease/Significant Fibrosis,” Journal of Affective Disorders 329 (2023): 184–191. 36841305 10.1016/j.jad.2023.02.101

[7] A. B. Nasir , S. Zouridis , P. Aspichueta , P. Manka , and W. K. Syn , “Relationship Between Depression and Chronic Liver Disease: Potential Role of Antidepressants in Modulating Liver Fibrosis,” American Journal of the Medical Sciences (2025): S0002‐9629(25)01214‐5, 10.1016/j.amjms.2025.07.018. 41043608

[8] S. M. Zhou , J. W. Li , J. P. Liu , et al., “Depressive Symptom as a Risk Factor for Cirrhosis in Patients With Primary Biliary Cholangitis: Analysis Based on Lasso‐Logistic Regression and Decision Tree Models,” Brain and Behavior 14, no. 8 (2024): e3639. 39099389 10.1002/brb3.3639 PMC11298689

[9] C. Laske , M. Zank , R. Klein , et al., “Autoantibody Reactivity in Serum of Patients With Major Depression, Schizophrenia and Healthy Controls,” Psychiatry Research 158, no. 1 (2008): 83–86. 18096244 10.1016/j.psychres.2006.04.023

[10] Y. Chen , T. Jiang , P. Chen , et al., “Emerging Tendency Towards Autoimmune Process in Major Depressive Patients: A Novel Insight From Th17 Cells,” Psychiatry Research 188, no. 2 (2011): 224–230. 21129782 10.1016/j.psychres.2010.10.029

[11] H. Gronbaek , M. Kreutzfeldt , K. Kazankov , et al., “Single‐Centre Experience of the Macrophage Activation Marker Soluble (s)CD163 ‐ Associations With Disease Activity and Treatment Response in Patients With Autoimmune Hepatitis,” Alimentary Pharmacology & Therapeutics 44, no. 10 (2016): 1062–1070. 27679428 10.1111/apt.13801

[12] M. Bartneck , V. Fech , J. Ehling , et al., “Histidine‐Rich Glycoprotein Promotes Macrophage Activation and Inflammation in Chronic Liver Disease,” Hepatology 63, no. 4 (2016): 1310–1324. 26699087 10.1002/hep.28418

[13] X. Yu , P. Lan , X. Hou , et al., “HBV Inhibits LPS‐Induced NLRP3 Inflammasome Activation and IL‐1beta Production via Suppressing the NF‐kappaB Pathway and ROS Production,” Journal of Hepatology 66, no. 4 (2017): 693–702. 28027970 10.1016/j.jhep.2016.12.018

[14] M. Iwata , K. T. Ota , X. Y. Li , et al., “Psychological Stress Activates the Inflammasome via Release of Adenosine Triphosphate and Stimulation of the Purinergic Type 2X7 Receptor,” Biological Psychiatry 80, no. 1 (2016): 12–22. 26831917 10.1016/j.biopsych.2015.11.026

[15] F. N. Kaufmann , A. P. Costa , G. Ghisleni , et al., “NLRP3 Inflammasome‐Driven Pathways in Depression: Clinical and Preclinical Findings,” Brain, Behavior, and Immunity 64 (2017): 367–383. 28263786 10.1016/j.bbi.2017.03.002

[16] M. L. Wong , A. Inserra , M. D. Lewis , et al., “Inflammasome Signaling Affects Anxiety‐ and Depressive‐Like Behavior and Gut Microbiome Composition,” Molecular Psychiatry 21, no. 6 (2016): 797–805. 27090302 10.1038/mp.2016.46 PMC4879188

[17] G. Xu , M. Yuan , H. He , et al., “NLRP3‐Mediated Trained Immunity of Microglia Is Involved in the Recurrence‐Like Episode of Depressive Disorders,” Molecular Psychiatry (2025), 10.1038/s41380-025-03344-y. 41249553

[18] E. Alcocer‐Gomez , N. Casas‐Barquero , M. R. Williams , et al., “Antidepressants Induce Autophagy Dependent‐NLRP3‐Inflammasome Inhibition in Major Depressive Disorder,” Pharmacological Research 121 (2017): 114–121. 28465217 10.1016/j.phrs.2017.04.028

[19] K. K. Jia , Y. J. Zheng , Y. X. Zhang , et al., “Banxia‐Houpu Decoction Restores Glucose Intolerance in CUMS Rats Through Improvement of Insulin Signaling and Suppression of NLRP3 Inflammasome Activation in Liver and Brain,” Journal of Ethnopharmacology 209 (2017): 219–229. 28782622 10.1016/j.jep.2017.08.004

[20] K. K. Jia , S. M. Pan , H. Ding , et al., “Chaihu‐Shugan San Inhibits Inflammatory Response to Improve Insulin Signaling in Liver and Prefrontal Cortex of CUMS Rats With Glucose Intolerance,” Biomedicine & Pharmacotherapy 103 (2018): 1415–1428. 29864926 10.1016/j.biopha.2018.04.171

[21] K. K. Jia , H. Ding , H. W. Yu , T. J. Dong , Y. Pan , and L. D. Kong , “Huanglian‐Wendan Decoction Inhibits NF‐kappaB/NLRP3 Inflammasome Activation in Liver and Brain of Rats Exposed to Chronic Unpredictable Mild Stress,” Mediators of Inflammation 2018 (2018): 3093516. 29853787 10.1155/2018/3093516 PMC5949167

[22] F. L. Shi , S. T. Ni , S. Q. Luo , et al., “Dimethyl Fumarate Ameliorates Autoimmune Hepatitis in Mice by Blocking NLRP3 Inflammasome Activation,” International Immunopharmacology 108 (2022): 108867. 35605433 10.1016/j.intimp.2022.108867

[23] G. A. Mohamed , S. R. M. Ibrahim , D. S. El‐Agamy , et al., “Cucurbitacin E Glucoside Alleviates Concanavalin A‐Induced Hepatitis Through Enhancing SIRT1/Nrf2/HO‐1 and Inhibiting NF‐kB/NLRP3 Signaling Pathways,” Journal of Ethnopharmacology 292 (2022): 115223. 35354089 10.1016/j.jep.2022.115223

[24] G. Liu , W. Zhao , J. Bai , J. Cui , H. Liang , and B. Lu , “Formononetin Protects Against Concanavalin‐A‐Induced Autoimmune Hepatitis in Mice Through Its Anti‐Apoptotic and Anti‐Inflammatory Properties,” Biochemistry and Cell Biology 99, no. 2 (2021): 231–240. 33749318 10.1139/bcb-2020-0197

[25] J. Luan , X. Zhang , S. Wang , et al., “NOD‐Like Receptor Protein 3 Inflammasome‐Dependent IL‐1beta Accelerated ConA‐Induced Hepatitis,” Frontiers in Immunology 9 (2018): 758. 29692782 10.3389/fimmu.2018.00758 PMC5902503

[26] Y. Wei , Y. Li , L. Yan , et al., “Alterations of Gut Microbiome in Autoimmune Hepatitis,” Gut 69, no. 3 (2020): 569–577. 31201284 10.1136/gutjnl-2018-317836

[27] B. Li , X. Liang , Y. Li , et al., “Tryptophan Catabolites From Microbiota Ameliorate Immune‐Mediated Hepatitis Through Activating Aryl Hydrocarbon Receptor of T Cells,” Gut Microbes 17, no. 1 (2025): 2557979. 40995824 10.1080/19490976.2025.2557979 PMC12477874

[28] E. D. Hu , D. Z. Chen , J. L. Wu , et al., “High Fiber Dietary and Sodium Butyrate Attenuate Experimental Autoimmune Hepatitis Through Regulation of Immune Regulatory Cells and Intestinal Barrier,” Cellular Immunology 328 (2018): 24–32. 29627063 10.1016/j.cellimm.2018.03.003

[29] G. Mieli‐Vergani , D. Vergani , A. J. Czaja , et al., “Autoimmune Hepatitis,” Nature Reviews Disease Primers 4 (2018): 18017. 10.1038/nrdp.2018.17 29644994

[30] C. Willyard , “How Gut Microbes Could Drive Brain Disorders,” Nature 590, no. 7844 (2021): 22–25. 33536656 10.1038/d41586-021-00260-3

[31] H. Zhang , X. Qin , H. Song , et al., “A Peripheral Mechanism of Depression: Disturbed Intestinal Epithelial Per2 Gene Expression Causes Depressive Behaviors in Mice With Circadian Rhythm Disruption via Gut Barrier Damage and Microbiota Dysbiosis,” Advanced Science 12, no. 43 (2025): e01818. 40847793 10.1002/advs.202501818 PMC12631932

[32] W. Hao , Q. Ma , L. Wang , et al., “Gut Dysbiosis Induces the Development of Depression‐Like Behavior Through Abnormal Synapse Pruning in Microglia‐Mediated by Complement C3,” Microbiome 12, no. 1 (2024): 34. 38378622 10.1186/s40168-024-01756-6 PMC10877840

[33] B. R. Stevens , R. Goel , K. Seungbum , et al., “Increased Human Intestinal Barrier Permeability Plasma Biomarkers Zonulin and FABP2 Correlated With Plasma LPS and Altered Gut Microbiome in Anxiety or Depression,” Gut 67, no. 8 (2018): 1555–1557. 10.1136/gutjnl-2017-314759 PMC5851874 28814485

[34] P. Zheng , B. Zeng , C. Zhou , et al., “Gut Microbiome Remodeling Induces Depressive‐Like Behaviors Through a Pathway Mediated by the Host's Metabolism,” Molecular Psychiatry 21, no. 6 (2016): 786–796. 27067014 10.1038/mp.2016.44

[35] Y. Zhang , R. Huang , M. Cheng , et al., “Gut Microbiota From NLRP3‐Deficient Mice Ameliorates Depressive‐Like Behaviors by Regulating Astrocyte Dysfunction via circHIPK2,” Microbiome 7, no. 1 (2019): 116. 31439031 10.1186/s40168-019-0733-3 PMC6706943

[36] L. Liao , K. M. Schneider , E. J. C. Galvez , et al., “Intestinal Dysbiosis Augments Liver Disease Progression via NLRP3 in a Murine Model of Primary Sclerosing Cholangitis,” Gut 68, no. 8 (2019): 1477–1492. 30872395 10.1136/gutjnl-2018-316670

[37] A. W. Lohse , O. Chazouilleres , G. Dalekos , et al., “EASL Clinical Practice Guidelines: Autoimmune Hepatitis,” Journal of Hepatology 63, no. 4 (2015): 971–1004. 26341719 10.1016/j.jhep.2015.06.030

[38] L. Muratori , A. W. Lohse , and M. Lenzi , “Diagnosis and Management of Autoimmune Hepatitis,” BMJ 380 (2023): e070201. 36746473 10.1136/bmj-2022-070201

[39] J. Lundberg , T. Cars , S. A. Loov , et al., “Association of Treatment‐Resistant Depression With Patient Outcomes and Health Care Resource Utilization in a Population‐Wide Study,” JAMA Psychiatry 80, no. 2 (2023): 167–175. 36515938 10.1001/jamapsychiatry.2022.3860 PMC9856735

[40] S. Takamoto , K. Nakamura , M. Yoneda , and I. Makino , “Gender‐Related Differences in Concanavalin A‐Induced Liver Injury and Cytokine Production in Mice,” Hepatology Research 27, no. 3 (2003): 221–229. 14585399 10.1016/s1386-6346(03)00263-8

[41] L. L. Liu , J. M. Li , W. J. Su , B. Wang , and C. L. Jiang , “Sex Differences in Depressive‐Like Behaviour May Relate to Imbalance of Microglia Activation in the Hippocampus,” Brain, Behavior, and Immunity 81 (2019): 188–197. 31181346 10.1016/j.bbi.2019.06.012

[42] A. Walther , J. Breidenstein , and R. Miller , “Association of Testosterone Treatment With Alleviation of Depressive Symptoms in Men: A Systematic Review and Meta‐Analysis,” JAMA Psychiatry 76, no. 1 (2019): 31–40. 30427999 10.1001/jamapsychiatry.2018.2734 PMC6583468

[43] S. U. Seo , N. Kamada , R. Munoz‐Planillo , et al., “Distinct Commensals Induce Interleukin‐1beta via NLRP3 Inflammasome in Inflammatory Monocytes to Promote Intestinal Inflammation in Response to Injury,” Immunity 42, no. 4 (2015): 744–755. 25862092 10.1016/j.immuni.2015.03.004 PMC4408263

[44] N. Mahmoud , M. F. Hegazy , W. Wadie , et al., “Naphthoquinone Derivatives as P‐Glycoprotein Inducers in Inflammatory Bowel Disease: 2D Monolayers, 3D Spheroids, and In Vivo Models,” Pharmacological Research 179 (2022): 106233. 35462013 10.1016/j.phrs.2022.106233

[45] S. Gong , J. Zheng , J. Zhang , et al., “Taxifolin Ameliorates Lipopolysaccharide‐Induced Intestinal Epithelial Barrier Dysfunction via Attenuating NF‐Kappa B/MLCK Pathway in a Caco‐2 Cell Monolayer Model,” Food Research International 158 (2022): 111502. 35840209 10.1016/j.foodres.2022.111502

[46] M. Cristina Lopes do Carmo , I. Mateus Martins , A. Elisa Ramos Magalhaes , M. Roberto Marostica Junior , and J. Alves Macedo , “Passion Fruit (*Passiflora edulis*) Leaf Aqueous Extract Ameliorates Intestinal Epithelial Barrier Dysfunction and Reverts Inflammatory Parameters in Caco‐2 Cells Monolayer,” Food Research International 133 (2020): 109162. 32466926 10.1016/j.foodres.2020.109162

[47] R. Snijders , M. K. Janik , M. Mund , et al., “Health‐Related Quality of Life Is Impaired in People With Autoimmune Hepatitis: Results of a Multicentre Cross‐Sectional Study Within the European Reference Network,” Hepatology 82, no. 5 (2025): 1058–1072. 39970194 10.1097/HEP.0000000000001271

[48] B. H. Mullish , M. S. Kabir , M. R. Thursz , and A. Dhar , “Review Article: Depression and the Use of Antidepressants in Patients With Chronic Liver Disease or Liver Transplantation,” Alimentary Pharmacology & Therapeutics 40, no. 8 (2014): 880–892. 25175904 10.1111/apt.12925

[49] H. Louis , A. Le Moine , E. Quertinmont , et al., “Repeated Concanavalin A Challenge in Mice Induces an Interleukin 10‐Producing Phenotype and Liver Fibrosis,” Hepatology 31, no. 2 (2000): 381–390. 10655261 10.1002/hep.510310218

[50] E. S. Wohleb , T. Franklin , M. Iwata , and R. S. Duman , “Integrating Neuroimmune Systems in the Neurobiology of Depression,” Nature Reviews Neuroscience 17, no. 8 (2016): 497–511. 27277867 10.1038/nrn.2016.69

[51] Y. Li , R. Zhang , Y. Wang , et al., “Hepatitis E Virus Infection Remodels the Mature tRNAome in Macrophages to Orchestrate NLRP3 Inflammasome Response,” Proceedings of the National Academy of Sciences of the United States of America 120, no. 25 (2023): e2304445120. 37307479 10.1073/pnas.2304445120 PMC10288653

[52] Y. Li , P. Yu , A. L. Kessler , et al., “Hepatitis E Virus Infection Activates NOD‐Like Receptor Family Pyrin Domain‐Containing 3 Inflammasome Antagonizing Interferon Response but Therapeutically Targetable,” Hepatology 75, no. 1 (2022): 196–212. 34392558 10.1002/hep.32114 PMC9299901

[53] V. T. Kronsten , T. H. Tranah , C. Pariante , and D. L. Shawcross , “Gut‐Derived Systemic Inflammation as a Driver of Depression in Chronic Liver Disease,” Journal of Hepatology 76, no. 3 (2022): 665–680. 34800610 10.1016/j.jhep.2021.11.008

[54] K. Gronke , M. Nguyen , H. Fuhrmann , et al., “Translocating Gut Pathobiont *Enterococcus gallinarum* Induces T(H)17 and IgG3 Anti‐RNA‐Directed Autoimmunity in Mouse and Human,” Science Translational Medicine 17, no. 784 (2025): eadj6294. 39908347 10.1126/scitranslmed.adj6294

[55] N. Nakamoto , N. Sasaki , R. Aoki , et al., “Gut Pathobionts Underlie Intestinal Barrier Dysfunction and Liver T Helper 17 Cell Immune Response in Primary Sclerosing Cholangitis,” Nature Microbiology 4, no. 3 (2019): 492–503. 10.1038/s41564-018-0333-1 30643240

[56] C. Roomruangwong , B. Kanchanatawan , S. Sirivichayakul , et al., “IgM‐Mediated Autoimmune Responses to Oxidative Specific Epitopes, but Not Nitrosylated Adducts, Are Significantly Decreased in Pregnancy: Association With Bacterial Translocation, Perinatal and Lifetime Major Depression and the Tryptophan Catabolite (TRYCAT) Pathway,” Metabolic Brain Disease 32, no. 5 (2017): 1571–1583. 28600633 10.1007/s11011-017-0040-2

[57] A. D. Frolkis , I. A. Vallerand , A. A. Shaheen , et al., “Depression Increases the Risk of Inflammatory Bowel Disease, Which May Be Mitigated by the Use of Antidepressants in the Treatment of Depression,” Gut 68, no. 9 (2019): 1606–1612. 30337374 10.1136/gutjnl-2018-317182

[58] A. A. Shaheen , G. G. Kaplan , W. Almishri , et al., “The Impact of Depression and Antidepressant Usage on Primary Biliary Cholangitis Clinical Outcomes,” PLoS One 13, no. 4 (2018): e0194839. 29617396 10.1371/journal.pone.0194839 PMC5884515

[59] W. Almishri , A. A. Shaheen , K. A. Sharkey , and M. G. Swain , “The Antidepressant Mirtazapine Inhibits Hepatic Innate Immune Networks to Attenuate Immune‐Mediated Liver Injury in Mice,” Frontiers in Immunology 10 (2019): 803. 31031775 10.3389/fimmu.2019.00803 PMC6474187

