# Supplementary material for: Depression Aggravates Immune‐Mediated Hepatitis Through NLRP3 Overactivation Induced by Intestinal Microbiota
Source: CNS Neurosci Ther. 2026 Jan 8;32(1):e70743. doi: 10.1002/cns.70743 (PMC12780859; doi:10.1002/cns.70743)
Supplement: Supplementary file 1 — Data S1: The Data S1 includes Supporting Informations and Methods, Supplementary Figures, and Supplementary Tables. Figure S1: Assessment of depressive‐like behaviors in mice. Figure S2: Depression promotes the activation of NLRP3 in immune‐mediated hepatitis. Figure S3: A highly specific NLRP3 inhibitor MCC950 exerts protective effects in mice. Figure S4: Fecal microbiota transplantation from patients with MDD induces adverse effects in recipient mice. Figure S5: Mirtazapine exerts protective effects by alleviating the disruption of intestinal barrier and the overactivation of NLRP3 in mice. Figure S6: Translocation of gut‐derived L. formosensis drives hepatic NLRP3 overactivation and liver injury in mice. Table S1: Comparison of general characteristics and laboratory parameters between patients with AIH and healthy controls. Table S2: Comparison of general characteristics and laboratory parameters between AIH patients with and without depression. Table S3: Comparison of general characteristics and laboratory parameters between AIH patients with and without cirrhosis. Table S4: Clinical characteristics of participants included in the PCR‐based analysis of hepatic NLRP3 pathway expression. [file CNS-32-e70743-s001.docx]

**Supplementary Material for**

**Depression aggravates** **immune-mediated hepatitis through NLRP3 overactivation induced by** **intestinal microbiota**

Simin Zhou^1^ | Liping Guo^1^ | Nian Chen^1^ | Haifeng Liu^1^ | Xin Liu^1^ | Jiwen Li^1^ | Shijing Dong^1^ | Jiangpeng Liu^1^ | Xiaoyi Wang^1^ | Ying Ran^1^ | Man Liu^1^ | Hongyu Chu^1^ | Yanni Li^1^ | Hui Yang^1^ | Jingwen Zhao^1^ | Lu Zhou^1^

^1^Department of Gastroenterology and Hepatology, Tianjin Medical University General Hospital, Tianjin, China

**Correspondence:** Lu Zhou (lzhou01@tmu.edu.cn) | Jingwen Zhao (jingwenzhao@tmu.ed.cn)

Department of Gastroenterology and Hepatology, General Hospital, Tianjin Medical University, Tianjin 300052, China

Tel/Fax: +86-022-60362608

**This file includes:**

Supplementary Materials and Methods

Figures S1-S6

Tables S1-S4

**Supplementary Materials and Methods**

**Chronic unpredictable mild stress (CUMS)-induced depressive-like behaviors in mice**

The CUMS procedure was performed according to a previous study [1]. In brief, CUMS mice, maintained in individual cages, were exposed to a random stressor once a day during the course of 8 weeks. No same stressor was applied in succession throughout the period. A variety of mild stressors were involved: (i) 24 h of food deprivation, (ii)24 h of water deprivation, (iii) 12 h of damp sawdust, (iv) 2 h of restraint stress, (v) 30 min of cage rotation, (vi) reversed light/dark cycle, (vii)12 h of empty cage tilt at 45 °C and (viii) 1 h of cold stress at 4 °C.

**Concanavalin A (ConA)-induced liver fibrosis in mice**

Mice were intravenously injected with ConA (15 mg/kg; Cat. No. C8110, Solarbio, Beijing, China) once a week during the course of 8 weeks to induce liver fibrosis (repeated ConA group). To investigate whether CUMS intervention could promote liver fibrosis induced by repeated ConA stimulation, CUMS processing was daily imposed on repeated ConA-treated mice (repeated ConA mice with CUMS intervention group). Mice were randomly allocated into 4 groups (10 in each group): Control, CUMS, repeated ConA and repeated ConA mice with CUMS intervention. Mice were sacrificed 16 h after the last injection of ConA.

**Mirtazapine and MCC950 treatment in mice**

CUMS mice were intraperitoneally injected with mirtazapine (20 mg/kg, dissolved in DMSO; Cat. No. HY-B0352, Med Chem Express, Monmouth Junction, USA) or MCC950 (20 mg/kg, dissolved in DMSO; Cat. No. abs810111, Absin, Shanghai, China) once a day during the last 2 weeks of the CUMS intervention. Mice were intravenously injected with ConA (15 mg/kg; Cat. No. C8110, Solarbio, Beijing, China) 2 h after the last injection of mirtazapine or MCC950 and sacrificed 16 h after ConA injection. We randomly divided the mice into 6 groups (10 in each group): Control, CUMS, ConA, ConA mice with CUMS intervention, ConA mice with CUMS and mirtazapine intervention, ConA mice with CUMS and MCC950 intervention.

**Isolation of murine peritoneal macrophages**

Female C57BL/6 mice (8 weeks old) were intraperitoneally injected with 1 mL of 3.85% Thioglycollate Medium Brewer Modified (Cat. No. 211716, BD Biosciences, Franklin Lakes, USA). Seventy-two hours after the injection, mice were anaesthetized and euthanized by cervical dislocation. The peritoneal cavity was gently lavaged with sterile phosphate-buffered saline (PBS) solution to maximize fluid recovery. After centrifugation, cells were cultured in Dulbecco’s modified Eagle’s medium (DMEM; Cat. No. 11965092, Gibco, Waltham, USA) containing 10% fetal bovine serum (Cat. No. 10099141, Gibco, Waltham, USA), 5000 units/mL penicillin and 5000 µg/mL streptomycin (Cat. No. 15070063, Gibco, Waltham, USA). Allow cells to attach for 6 h in a humidified incubator with 5% CO_2_ at 37 °C. After the non-adherent cells were discarded, the adherent peritoneal macrophages were isolated.

**Cell culture**

Caco-2 epithelial cell monolayers (BNCC 338148) were cultured in DMEM containing 10% fetal bovine serum, 5000 units/mL penicillin and 5000 µg/mL streptomycin. Cells were incubated at an atmosphere containing 5% CO_2_ at 37 °C. After pretreatment with mirtazapine (2, 5 and 10 µM) for 1 h, Caco-2 cells and isolated murine peritoneal macrophages were stimulated with lipopolysaccharide (LPS) (3 µg/mL; Cat. No. L8880, Solarbio, Beijing, China) for 12 h and 6 h, respectively.

**Flow cytometry analysis in mice**

Liver samples were digested in the Hank's balanced salt solution (Cat. No. H1045, Solarbio, Beijing, China) containing type IV collagenase (0.05%; Cat. No. C8160, Solarbio, Beijing, China) and DNase I (50 μg/mL; Cat. No. D8071, Solarbio, Beijing, China) at 37 °C for 30 min on a shaking incubator. The cell suspensions were filtered through 70 μm nylon cell strainers and further centrifuged. Then, the obtained cell pellet was resuspended in 30% Percoll (Cat. No. P8370, Solarbio, Beijing, China), gently overlaid onto 70% Percoll and further centrifuged. The white cell layers between 30% and 70% Percoll were mononuclear cells. Simultaneously, spleens were collected, ground and filtered. After removal of erythrocytes, purified mononuclear cells from the liver and spleen samples were collected. The isolated cells were incubated with a cocktail of fluorescently conjugated antibodies for 30 min at 4 °C in the dark: anti-CD45-FITC (2 μL; 553079, BD Biosciences, Franklin Lakes, USA), anti-CD11b-PE (2 μL; 557397, BD Biosciences, Franklin Lakes, USA), and anti-Ly6C-BV421 (2 μL; 562727, BD Biosciences, Franklin Lakes, USA). Data were acquired on LSRFortessa flow cytometry machine (BD Biosciences, Franklin Lakes, USA) and analyzed by FlowJo (BD Biosciences, Franklin Lakes, USA).

**Fecal microbiota transplantation**

Patients with major depressive disorder (MDD) enrolled in the fecal microbiota transplantation experiment should meet the following criteria: (i) without receiving any antidepressant drugs; (ii) without taking probiotics or antibiotics in the recent two months; (iii) without suffering from any autoimmune diseases or malignant tumors. Fecal microbiota transplantation (FMT) was performed in mice with fecal samples obtained from either MDD patients (n=5) or age- and sex-matched healthy controls (HC, n=5). Mice received broad-spectrum antibiotics (a cocktail of ampicillin [1 g/L; Cat. No. A8180, Solarbio, Beijing, China], neomycin [1 g/L; Cat. No. N8090, Solarbio, Beijing, China], metronidazole [1 g/L; Cat. No. IM0230, Solarbio, Beijing, China] and vancomycin [0.5 g/L; Cat. No. IV0030, Solarbio, Beijing, China]) in drinking water for 7 d. The antibiotic solution was supplemented with sweetener (Equal, 4 g/L) to overcome the metallic taste of metronidazole. After antibiotic discontinuation for 3 d, the FMT experiment was performed via oral gavage according to a previous study [2]. Mixed fecal samples were suspended with sterile PBS solution (1:5) and sequentially filtered through sterile filters with apertures of 2.0, 1.0, 0.5 and 0.25 mm. Following centrifugation, the supernatant was collected and administered to specific-pathogen free (SPF) mice via oral gavage according to the following regimen: daily for the first week, three times weekly during the second week, and weekly for the subsequent six weeks. Following the last gavage administration, mice were intravenously injected with ConA (15 mg/kg; Cat. No. C8110, Solarbio, Beijing, China) and sacrificed 16 h after ConA injection. Mice were randomly allocated into 5 groups (10 in each group): Control, FMT-HC, FMT-MDD, ConA + FMT-HC and ConA + FMT-MDD.

To confirm the existence of any bacteria in the extraintestinal organs, the liver, spleen and mesenteric lymph nodes (MLN) were carefully harvested and aerobically or anaerobically cultured on Columbia blood agar, deoxycholate hydrogen sulfide lactose agar, spirulina agar and MacConkey agar plates. Isolates were identified by 16S rRNA gene sequencing.

**Culture of *Lactococcus formosensis***

*L. formosensis* was cultured aerobically in deMan, Rogosa, and Sharpe (MRS) broth (Cat. No. M8540, Solarbio, Beijing, China) at 37 °C until reaching mid-logarithmic growth phase. Following centrifugation at 5000 g for 10 min, the supernatant was collected, filtered through 0.22 μm filters and serially diluted to three concentration gradients (1:25, 1:50 and 1:100). The isolated murine peritoneal macrophages were treated with the diluted supernatant for 24 h.

**Vaccine preparation**

*L. formosensis* was heat-killed at 110 °C for 10 min. Each vaccine contained 100 µg of heat-killed *L. formosensis*, 100 µg of polysorbate 80 (Cat. No. T8360, Solarbio, Beijing, China), 0.125 mg of aluminium phosphate (Cat. No. A800891, Macklin, Shanghai, China), and 295 µg of succinate (Cat. No. S8260, Solarbio, Beijing, China). The dosing scheme was performed according to a previous study [3].

***L. formosensis* monocolonization experiment**

Following antibiotic pretreatment regimen (a cocktail of ampicillin, neomycin, metronidazole, and vancomycin in drinking water) and a subsequent 3-day antibiotic withdrawal interval, mice were colonized via weekly oral gavage with 1 × 10^6^ colony-forming units of *L. formosensis* for 8 consecutive weeks. Before the first colonization, mice received the vaccine targeting *L. formosensis*-specific DNA via intramuscular injection weekly for 2 consecutive weeks. Mice were randomly allocated into four groups (n = 10 per group): Control group, vaccine-only group, *L. formosensis* monocolonized group and pre-vaccinated *L. formosensis* monocolonized group.

**Preparation of green fluorescent protein (GFP)-labelled *L. formosensis***

We fluorescently labeled *L. formosensis* strain with green fluorescent plasmid. A total of 5 μL of the green fluorescent plasmid was added into 90 μL of the competent cells. After incubated at 4 °C for 5 min, the cells were transformed by electroporation at 2 KV. Afterwards, they were coated with an erythromycin resistance plate and incubated at 37 °C overnight. Mice colonized with *L. formosensis* for 8 weeks were gavaged with GFP-labelled *L. formosensis*. After 48 h, liver and MLN were aseptically dissected and photographed by IVIS SPECTRUM (PE, Waltham, USA).

**Intestinal permeability assay**

Fluorescein isothiocyanate-dextran (FITC-D, 4000 MW; Cat. No. FD4, Sigma Aldrich, St. Louis, USA) was dissolved in PBS and administered to mice via oral gavage at a dose of 600 mg/kg body weight. Four hours after the administration of FITC-D, the blood samples were collected. The concentration of FITC-D was measured on the basis of the FITC-D standard curve.

**Intestinal microbiota analysis**

The 16S rRNA gene sequencing process was carried out by Novogene Institute (Beijing, China). Quantitative real-time polymerase chain reaction (PCR) products were purified with QIAquick gel extraction kit (Cat. No. 28704, QIAGEN, Hilden, Germany). SMRTbell Template Prep Kit (PacBio) was used in generating sequencing libraries. Amplicon libraries were quantified with Qubit@ 2.0 fluorometer (Thermo Scientific) and sequenced on a PacBio Sequel platform.

**PCR**

TRIzol^TM^ reagent (Cat. No. 15596018, Invitrogen, Carlsbad, USA) was applied to extract RNA from tissues or adherent cells. 1 μg of total RNA was used to synthesize cDNA by FastKing RT Kit (Cat. No. KR116, TIANGEN, Beijing, China). Quantitative real-time PCR was performed by using SYBR® Select Master Mix (Cat. No. 4472908, Thermo Fisher Scientific, Waltham, USA). Relative mRNA expression was counted on the basis of the fold-changes normalized to glyceraldehyde-3-phosphate dehydrogenase (*GAPDH*) with the method of 2^−ΔΔCt^.

Human primer sequences were listed below: GAPDH (Forward: 5′-CATCACTGCCACCCAGAAGACTG-3′, Reverse: 5′-ATGCCAGTGAGCTTCCCGTTCAG-3′); NLR family pyrin domain containing 3 (NLRP3) (Forward: 5′-GGACTGAAGCACCTGTTGTGCA-3′, Reverse: 5′-TCCTGAGTCTCCCAAGGCATTC-3′); Caspase 1 (CASP1) (Forward: 5′-GCCTGTTCCTGTGATGTGGAG-3′, Reverse: 5′-TGCCCACAGACATTCATACAGTTTC-3′); Interleukin 1 beta (IL1B) (Forward: 5′-GACCTGAGCACCTTCTTTCCCTTC-3′, Reverse: 5′-GCAGTTCAGTGATCGTACAGGTGC-3′). Murine primer sequences were listed as follows: Gapdh (Forward: 5′-GGAGAAACCTGCCAAGTATG-3′, Rev-erse: 5′-TGGGAGTTGCTGTTGAAGTC-3′); Actin alpha 2 (Acta2) (Forward: 5′-AGCCATCTTTCATTGGGATGG-3′, Reverse: 5′-CCCCTGACAGGACGTTGTTA-3′); Tissue inhibitor of metalloproteinase 1 (Timp1) (Forward:5′-GGCATCTGGCATCCTCTTGT-3′, Reverse: 5′-TTAGCATCCAGGTCCGAGTTG-3′); Collagen, type I, alpha 1 (Col1a1) (Forward: 5′-CCTCAGGGTATTGCTGGACAAC-3′, Reverse: 5′-CAGAAGGACCTTGTTTGCCAGG-3′); Nlrp3 (F-orward: 5′-ATCAACAGGCGAGACCTCTG-3′, Reverse: 5′-GTCCTCCTGGCATACCATAGA-3′); Casp1 (Forward: 5′-ACAAGGCACGGGACCTATG-3′, R-everse: 5′-TCCCAGTCAGTCCTGGAAATG-3′); Il1b (Forward: 5′-TGGACCTTCCAGGATGAGGACA-3′, Reverse: 5′-GTTCATCTCGGAGCCTGTAGTG-3′); Tight junction protein 1 (Tjp1) (Forward: 5′-GGGCCATCTCAACTCCTGTA-3′, Reverse: 5′-AGAAGGGCTGACGGGTAAAT-3′); Occludin (Ocln) (Forw-ard: 5′-TGAAAGTCCACCTCCTTACAGA-3′, Reverse: 5′-CCGGATAAAAAGAGTACGCTGG-3′).

**Western blot analysis**

Tissues were lysed with radio-immunoprecipitation assay (RIPA) lysis buffer (Cat. No. R0010, Solarbio, Beijing, China) containing 1 mM protein phosphatase inhibitor (Cat. No. P1260, Solarbio, Beijing, China). Protein concentration was determined using a bicinchoninic acid protein assay kit (Cat. No. PC0020, Solarbio, Beijing, China). Protein samples were separated by sodium dodecyl sulfate-polyacrylamide gel electrophoresis (SDS-PAGE) and further transferred to polyvinylidene fluoride (PVDF) membranes (Cat. No. 1620177, Bio-Rad Laboratories, Hercules, USA). After blocking with 5% skim milk, the membranes were incubated overnight at 4 °C with the following primary antibodies: rabbit anti-ZO1 (1:1000; ab276131, Abcam, Cambridge, UK), rabbit anti-OCLN (1:1000; ab167161, Abcam, Cambridge, UK), rabbit anti-NLRP3 (1:1000; 15101, CST, MA, USA), and rabbit anti-GAPDH (1:1000; 3683, CST, MA, USA). Following incubation, the membranes were washed three times with Tris-Buffered Saline with Tween 20 (TBST) and then incubated with horseradish peroxidase (HRP)-conjugated goat anti-rabbit IgG secondary antibody (1:3000; 7074, CST, MA, USA) for 1 h at room temperature. The bolts were exposed to the ChemiDoc^TM^ XRS^+^ Imaging System (BIO RAD, Hercules, USA) for visualization of protein bands. Densitometric analyses were performed using the NIH Image J Software.

**Enzyme-linked immunosorbent assay (ELISA)**

Serum levels of D-lactic acid (DLA) (Cat. No. F10011-A, Huyu Biotechnology, Shanghai, China) (standard range 60-1600 μg/L, inter-assay coefficiency %Coefficients of Variation [CV] = 10.5, intra-assay coefficiency %CV = 8.4), diamine oxidase (DAO) (Cat. No. F0560-A, Huyu Biotechnology, Shanghai, China) (standard range 6-250 pg/mL, inter-assay coefficiency %CV = 8.6, intra-assay coefficiency %CV = 6.5), fatty acid-binding protein-2 (FABP2) (Cat. No. F10367-A, Huyu Biotechnology, Shanghai, China) (standard range 0.5-12 ng/mL, inter-assay coefficiency %CV = 9.1, intra-assay coefficiency %CV = 7.3) and LPS (Cat. No. F1302-A, Huyu Biotechnology, Shanghai, China) (standard range 20-480 ng/L, inter-assay coefficiency %CV = 9.5, intra-assay coefficiency %CV = 7.9) were measured in human participants using ELISA kits according to the manufacturer’s instructions. LPS levels in murine serum were similarly determined with a murine-specific ELISA kit (Cat. No. SBJ-M0941, SenBeiJia Biotech, Nanjing, China) (standard range 10-300 ng/L).

**Histological analysis**

Formaldehyde-fixed liver tissues were paraffin-embedded and cut into 5µm sections. Procedures of hematoxylin and eosin (H&E), Sirius red and Masson staining were carried out according to standard procedures. The severity of inflammatory cell infiltration was graded using the 4-point scoring system described by Mann et al [4]. The pathological severity scoring was independently assessed by two professional pathologists. For immunofluorescence staining, tissue sections were incubated overnight at 4 °C with the following primary antibodies: rabbit anti-occludin (1:100; ab216327, Abcam, Cambridge, UK), rabbit anti-CD68 (1:100; ab213363, Abcam, Cambridge, UK), rabbit anti-F4/80 (1:100; PA1046, Bios Biological, Wuhan, China), and rabbit anti-NLRP3 (1:50; NBP2-12446, Novus Biologicals, Littleton, USA). The next day, slides were equilibrated to room temperature and incubated with the following fluorescent secondary antibodies for 1 h in the dark: Alexa Fluor 488-conjugated anti-rabbit IgG (1:500; ab150077, Abcam, Cambridge, UK) for Occludin and CD68, Alexa Fluor 594-conjugated anti-rabbit IgG (1:500; ab150080, Abcam, Cambridge, UK) for NLRP3, and FITC-conjugated anti-rabbit IgG (1:500; ab97050, Abcam, Cambridge, UK) for F4/80. 4, 6-Diamidino-2-phenylindole (Cat. No. S2110, Solarbio, Beijing, China) was finally applied to dye the nucleus. DM5000B (Leika, Wetzlar, Germany) was used in obtaining fluorescence photographs.

**Sucrose preference test**

Mice were allowed free access to 1% sucrose solution from two differing bottles during the first 24 h. In the subsequent 24 h, the mice could individually receive 1% sucrose and water from two different bottles. Bottle side preference was prevented by switching the positions of water and sucrose (left or right) every 6 h. The mice were deprived of water and food for the next 18 h and then had free access to 1% sucrose and water. Similarly, the positions of water and sucrose were interchanged every 2 h. Sucrose preference was calculated as the percentage of sucrose intake relative to the total amounts of water and sucrose intake.

**Open-field test**

Mice were gently placed in an open-field apparatus (50 × 50 × 40 cm). The bottom of the box was divided into 16 squares on average, with 4 squares in the center being the central zone. After adaptation, mice were allowed to explore freely for 10 min. The total motion distance and the frequency of entering into the central area were construed as indicators of depressive-like behaviors. The arena was thoroughly cleaned with 75% ethanol after each test session. Spontaneous activities were recorded by a video tracking system (SuperMaze, Shanghai, China).

**References**

1. B. Jiang, H. Wang, J. L. Wang, et al., “Hippocampal Salt-Inducible Kinase 2 Plays a Role in Depression via the CREB-Regulated Transcription Coactivator 1-cAMP Response Element Binding-Brain-Derived Neurotrophic Factor Pathway,” *Biol Psychiatry* 85, no. 8 (2019): 650-666.

2. L. Li, X. Li, W. Zhong, et al., “Gut microbiota from colorectal cancer patients enhances the progression of intestinal adenoma in Apc(min/+) mice,” *EBioMedicine* 48, no. (2019): 301-315.

3. S. Manfredo Vieira, M. Hiltensperger, V. Kumar, et al., “Translocation of a gut pathobiont drives autoimmunity in mice and humans,” *Science* 359, no.6380 (2018): 1156-1161.

4. P. C. Mann, J. Vahle, C. M. Keenan, et al., “International harmonization of toxicologic pathology nomenclature: an overview and review of basic principles,” *Toxicol Pathol* 40, no. 4 Suppl (2012): 7S-13S.

**Supplementary Figures**

**
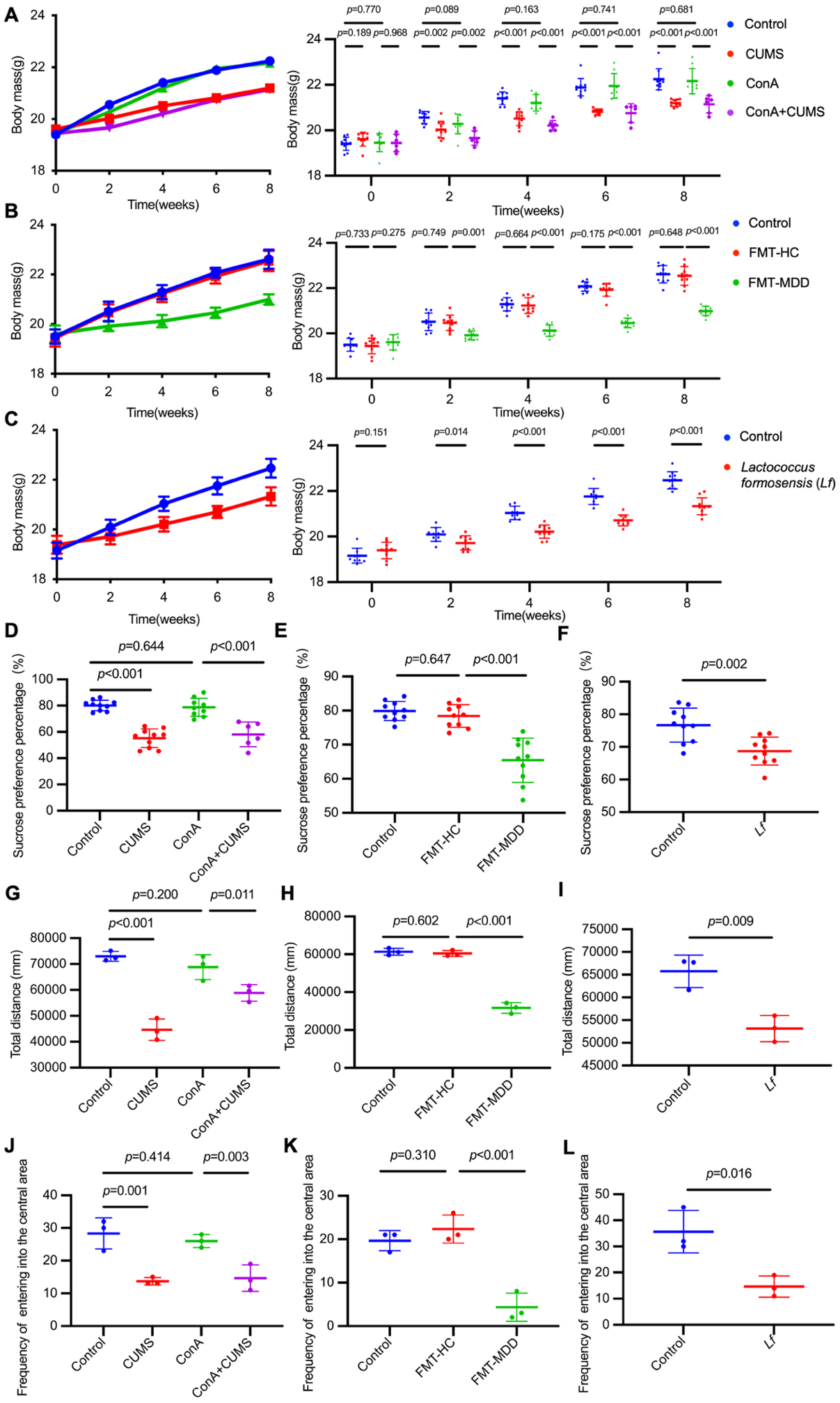
**

**Figure S1** | Assessment of depressive-like behaviors in mice. (A–C) Weight loss in chronic unpredictable mild stress (CUMS) model (A), fecal microbiota transplantation (FMT) model (B) and *Lactococcus formosensis* monocolonization model (C). A: Df = 3, n = 10, 10, 9, 6. Week 0: F = 0.689, week 2: F = 9.017, week 4: F = 27.932, week 6: F = 24.281, week 8: F = 17.870. B: Df = 2, n = 10. Week 0: F = 0.652, week 2: F = 11.068, week 4: F = 47.993, week 6: F = 143.443, week 8: F = 69.595. C: Df = 18, n = 10. Week 0: t = -1.499, week 2: t = 2.722, week 4: t = 6.354, week 6: t = 7.828, week 8: t = 6.775. (D–F) Sucrose preference in CUMS model (D), FMT model (E) and *L. formosensis* monocolonization model (F). Sucrose preference was calculated as the percentage of sucrose intake relative to total amount of water and sucrose intake. The evaluation of depression-like behavior by open-field test. (G–I) Total distances in CUMS model (G), FMT model (H) and *L. formosensis* monocolonization model (I). (J–L) Frequency of entering into the central area in CUMS model (J), FMT model (K) and *L. formosensis* monocolonization model (L). D: F = 34.517, Df = 3, n = 10, 10, 9, 6. E: F = 31.137, Df = 2, n = 10. F: t = 3.738, Df = 18, n = 10. G: F = 35.231, Df = 3, n = 3. H: F = 187.984, Df = 2, n = 3. I: t = 4.769, Df = 4, n = 3. J: F = 15.657, Df = 3, n = 3. K: F = 32.667, Df = 2, n = 3. L: t = 4.001, Df = 4, n = 3. All the values are shown as mean ± standard deviation (SD). Significantly different indicators between groups were identified by unpaired t-test (F, I, L) and one-way analysis of variance (ANOVA) with Fisher’s least significant difference (LSD) post hoc test (A–D, G–H, J–K) and one-way ANOVA with Tamhane’s T2 post hoc test (E).

**
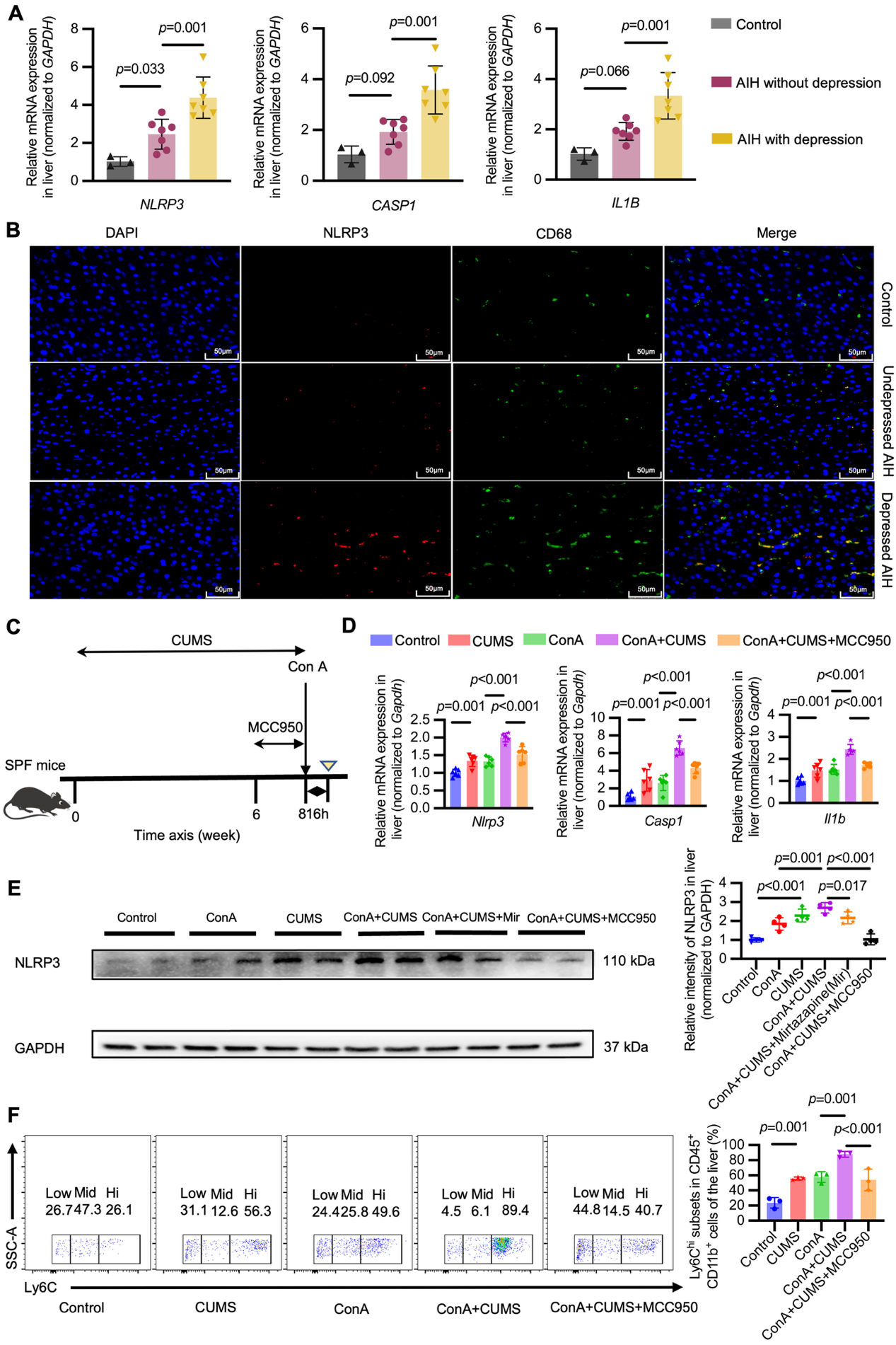
**

**Figure S2** | Depression promotes the activation of NLR family pyrin domain containing 3 (NLRP3) in immune-mediated hepatitis. (A) Polymerase chain reaction (PCR) analysis of *NLRP3*, caspase-1 (*CASP1*), and interleukin 1 beta (*IL1B*) relative to glyceraldehyde-3-phosphate dehydrogenase (*GAPDH*) in liver biopsies from autoimmune hepatitis (AIH) patients with/without depression and control group. F = 17.239, 16.740, 15.774; all Df = 2; n = 3, 7, 7. (B) Double immunofluorescence staining with CD68 and NLRP3 in liver sections from AIH patients with/without depression and control group. Scale bars, 50 μm. (C) Study design: CUMS-induced depression mice were intraperitoneally injected with MCC950 daily during the last 2 weeks. Two hours after the last injection of MCC950, mice were intravenously injected with Concanavalin A (ConA). The mice were sacrificed 16 h after ConA injection (yellow triangle). (D) PCR analysis of *Nlrp3*, *Casp1*, and *Il1b* relative to *Gapdh* in liver biopsies from the indicated groups. F = 34.244, 36.763, 33.638, all Df = 4, all n = 6. (E) Western blot analysis of NLRP3 relative to GAPDH in liver biopsies from the indicated groups. F = 23.577, Df = 5, n = 4. (F) The proportions of CD45^+^CD11b^+^Ly6C^hi^ cells among CD45^+^CD11b^+^ cells in the liver were quantified by flow cytometry analysis. F = 24.011, Df = 4, n = 3. All the values are shown as mean ± SD. Statistical significance was identified by one-way ANOVA with LSD post hoc test.

**
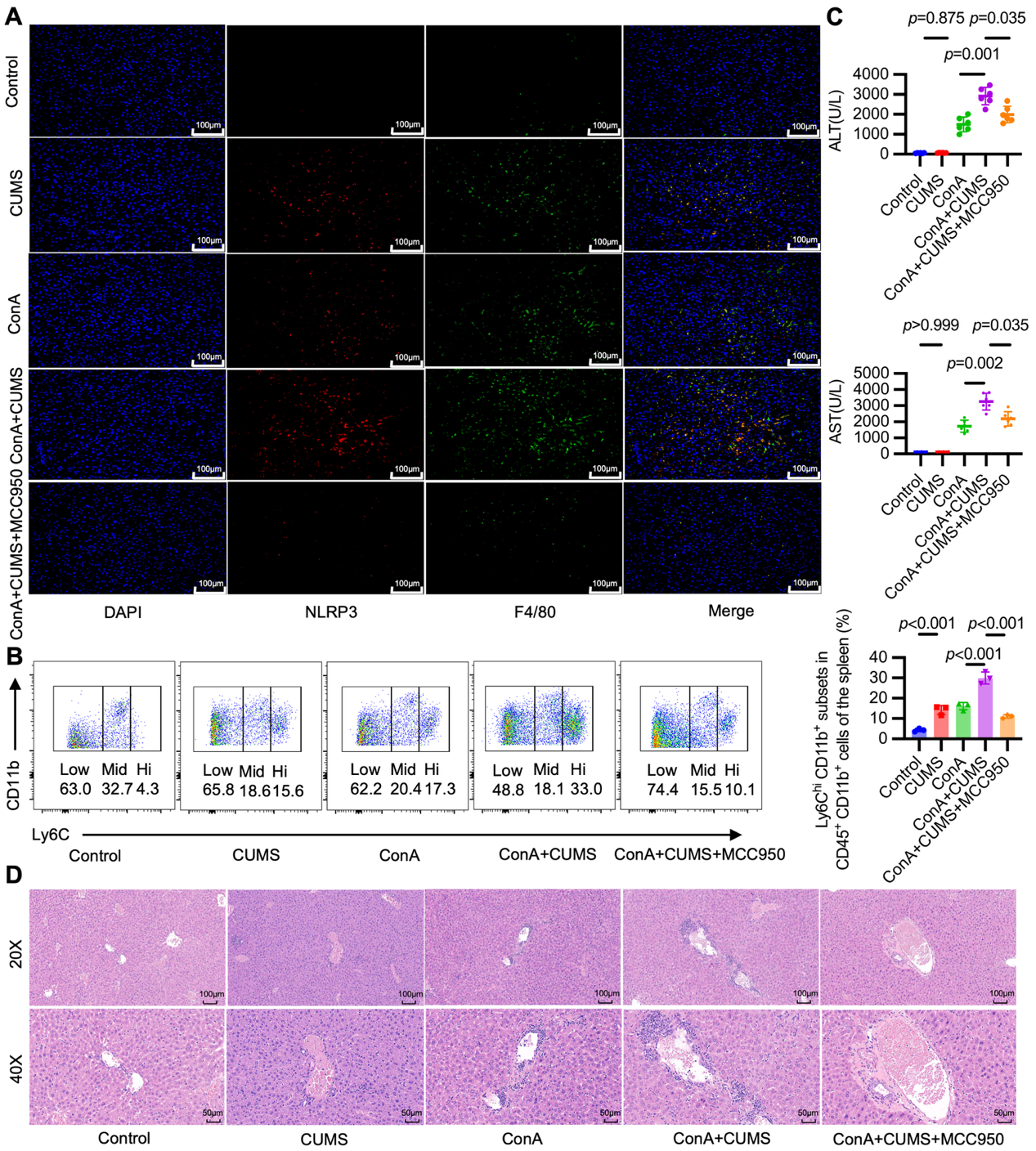
 Figure S3** | A highly specific NLRP3 inhibitor MCC950 exerts protective effects in mice. (A) Double immunofluorescence staining with F4/80 and NLRP3 in liver tissues of mice. Scale bars, 100μm. (B) The proportions of CD45^+^CD11b^+^Ly6C^hi^ cells among CD45^+^CD11b^+^ cells in the spleen were quantified by flow cytometry analysis. F = 72.757, Df = 4, n = 3. (C) The serum levels of alanine aminotransferase (ALT) and aspartate aminotransferase (AST) in mice. F = 93.609, 90.361, Df = 4, n = 6. (D) Representative hematoxylin and eosin (H&E) staining images of liver tissues were shown (Scale bars: 100μm, 50μm). All the values are shown as mean ± SD. Statistical significance was identified by one-way ANOVA with LSD post hoc test (B) and Tamhane’s T2 post hoc test (C).


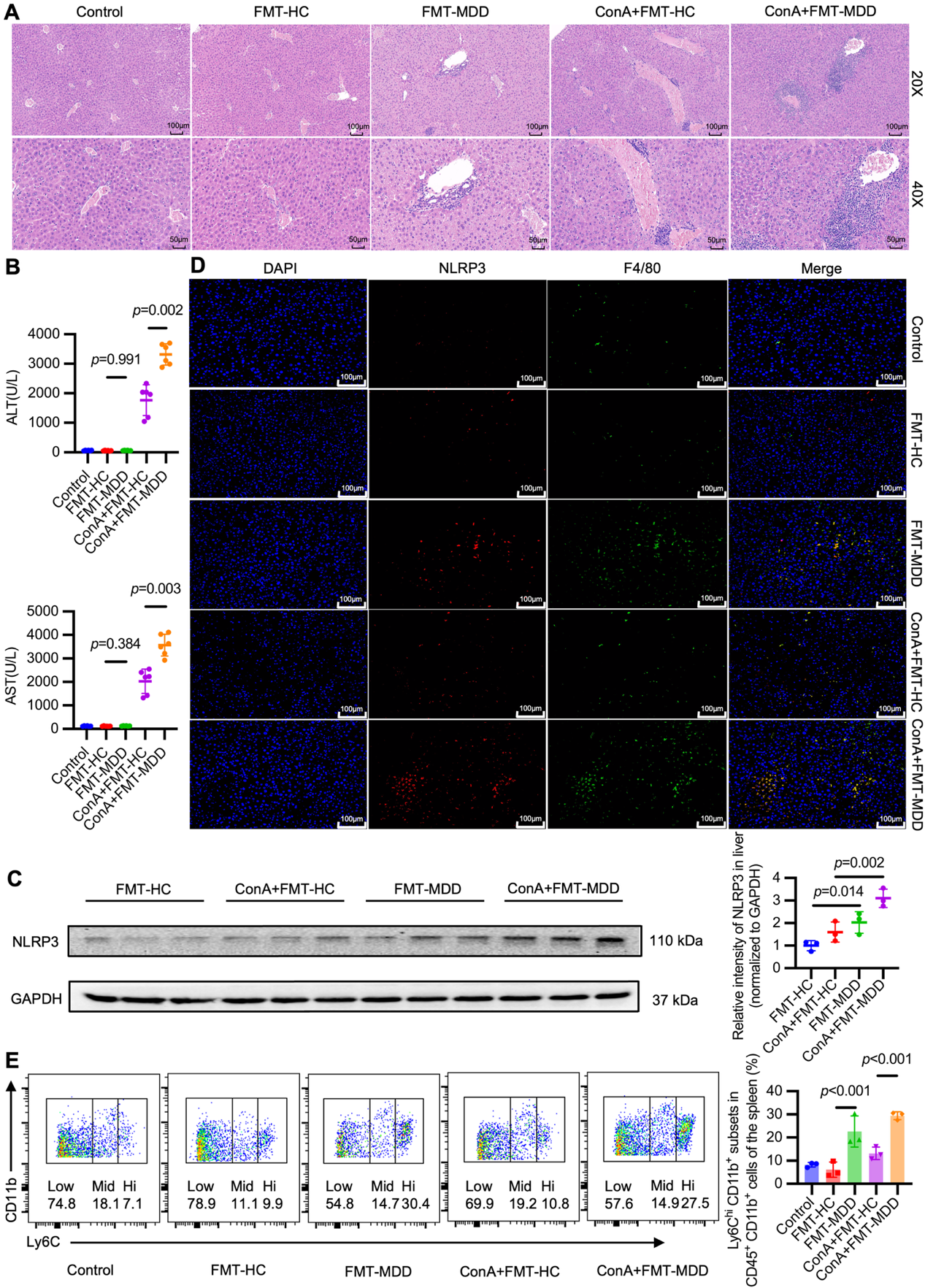


**Figure S4** | Fecal microbiota transplantation from patients with major depressive disorder (MDD) induces adverse effects in recipient mice. (A) Representative H&E staining images of liver tissues were shown (Scale bars: 100μm, 50μm). (B) The serum levels of ALT and AST in mice. F = 159.853, 151.428, Df = 4, n = 6. (C) Western blots analysis of NLRP3 relative to GAPDH in liver biopsies from the indicated groups. F = 14.451, Df = 3, n = 3. (D) Double immunofluorescence staining with F4/80 and NLRP3 in liver tissues of mice. Scale bars, 100μm. (E) The proportions of CD45^+^CD11b^+^Ly6C^hi^ cells among CD45^+^CD11b^+^ cells in the spleen were quantified by flow cytometry analysis. F = 21.619, Df = 4, n = 3. All the values are shown as mean ± SD. Statistical significance was identified by one-way ANOVA with LSD post hoc test (C, E) and Tamhane’s T2 post hoc test (B).

**
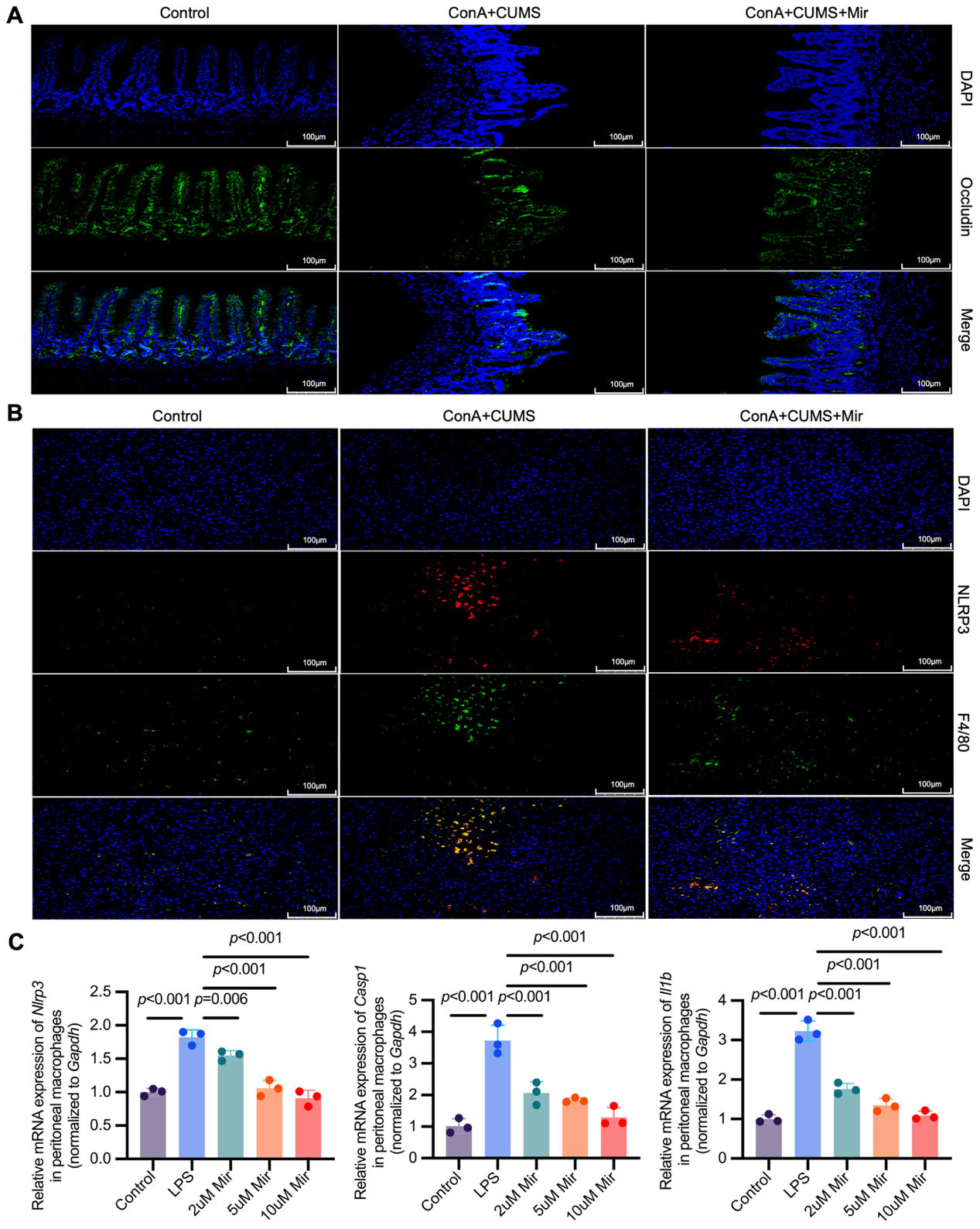
 Figure S5** | Mirtazapine exerts protective effects by alleviating the disruption of intestinal barrier and the overactivation of NLRP3 in mice. (A) Representative immunofluorescence staining with Occludin in the intestine. Scale bars, 100 μm. (B) Double immunofluorescence staining with F4/80 and NLRP3 in liver tissues. Scale bars, 100 μm. (C) PCR analysis of *Nlrp3*, *Casp1* and *Il1b* in LPS-treated murine peritoneal macrophages pretreated with mirtazapine in a concentration-dependent manner. F = 48.988, 32.089, 89.789, Df = 4, n = 3. All the values are presented as mean ± SD. Statistical significance was identified by one-way ANOVA with LSD post hoc test.

**
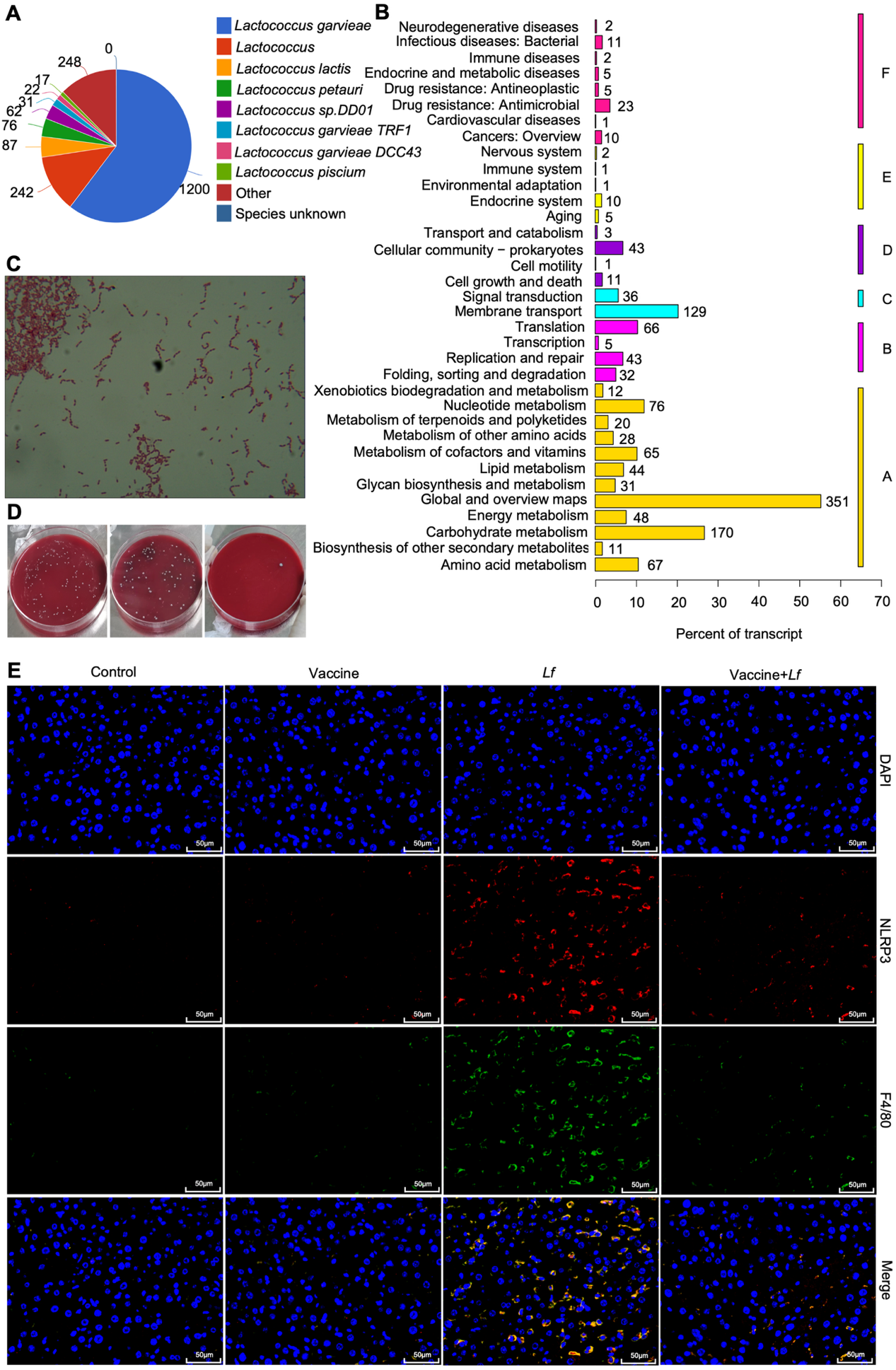
 Figure S6** | Translocation of gut-derived *L. formosensis* drives hepatic NLRP3 overactivation and liver injury in mice. (A) Species distribution of *Lactococcus.* (B) Transcript pathway classification by Kyoto Encyclopedia of Genes and Genomes (KEGG) using de novo analysis of *L. formosensis*. (C) Microscopic characteristics of *L. formosensis*. (D) *L. formosensis* could be grown from the liver of mice harboring microbiota from patients with major depressive disorder (left), liver of mice monocolonized with *L. formosensis* (middle) and mesenteric lymph nodes (MLN) of mice monocolonized with *L. formosensis* (right). (E) Double immunofluorescence staining with F4/80 and NLRP3 in liver tissues of mice monocolonized with *L. formosensis*. Scale bars, 50μm.

**Supplementary Tables**

**Table S1** | Comparison of general characteristics and laboratory parameters between patients with AIH and healthy controls.

|  | **Control**  **(n = 173)** | **Patients with AIH (n = 260)** | ***p-*value** |
| --- | --- | --- | --- |
| **General characteristics** |  |  |  |
| Age (median with IQR) | 58.0 (50.0-65.0) | 58.0 (49.0-64.0) | 0.633^†^ |
| Women (n, %) | 149 (86.1%) | 225 (86.5%) | 0.903^‡^ |
| **Laboratory parameters** |  |  |  |
| TP (median with IQR, g/L) | 76.0 (72.0-78.0) | 75.0 (71.0-79.0) | 0.119^†^ |
| ALB (median with IQR, g/L) | 46.0 (44.0-47.0) | 42.0 (40.0-44.0) | <0.001^†^ |
| GLO (median with IQR, g/L) | 30.0 (28.0-31.0) | 32.0 (30.0-35.0) | <0.001^†^ |
| ALT (median with IQR, U/L) | 19.0 (14.0-25.5) | 32.5 (27.0-39.8) | <0.001^†^ |
| AST (median with IQR, U/L) | 21.0 (15.0-27.0) | 34.0 (28.0-42.0) | <0.001^†^ |
| ALP (median with IQR, U/L) | 78.0 (65.0-90.0) | 97.0 (78.0-121.8) | <0.001^†^ |
| GGT (median with IQR, U/L) | 19.0 (13.0-27.5) | 35.0 (22.0-43.8) | <0.001^†^ |
| TB (median with IQR, μmol/L) | 9.8 (8.2-11.4) | 12.8 (11.0-15.0) | <0.001^†^ |
| DB (median with IQR, μmol/L) | 3.1 (2.3-4.1) | 3.6 (3.0-4.5) | <0.001^†^ |
| IgG (median with IQR, mg/dL) | - | 1490.0 (1340.0-1667.5) | - |
| IgM (median with IQR, mg/dL) | - | 124.5 (94.6-167.5) | - |
| ANA titer (*≥*1:160) (n, %) | - | 138 (53.1%) | - |
| C3 (median with IQR, mg/dL) | - | 99.5 (83.6-111.0) | - |
| C4 (median with IQR, mg/dL) | - | 20.1 (16.6-24.2) | - |
| WBC (median with IQR, *10^9^/L) | - | 5.2 (4.4-6.1) | - |
| Hb (mean ± SD, g/L) | - | 130.9 ± 14.0 | - |
| PLT (median with IQR, *10^9^/L) | - | 189.5 (151.3-220.0) | - |
| **Severity of depression** |  |  |  |
| Depression (n, %) | 26 (15.0%) | 106 (40.8%) | <0.001^‡^ |
| HAMD score (median with IQR) | 3.0 (2.0-5.0) | 7.0 (4.0-14.0) | <0.001^†^ |
| **Disease assessment** |  |  |  |
| Cirrhosis (n, %) | - | 90 (34.6%) | - |
| Extrahepatic AIDs (n, %) | - | 120 (46.2%) | - |
| Enlarged ALN (n, %) | - | 135 (51.9%) | - |

Note: Without depression: HAMD score < 8; With depression: HAMD score ≥ 8. ^†^: the statistic value was based on Mann-Whitney U test; ^‡^: the statistic value was based on chi-square test.

Abbreviations: AIH, autoimmune hepatitis; IQR, interquartile range; SD, standard deviation; TP, total protein; ALB, albumin; GLO, globulin; ALT, alanine aminotransferase; AST, aspartate aminotransferase; ALP, alkaline phosphatase; GGT, γ-glutamyl transpeptidase; TB, total bilirubin; DB, direct bilirubin; Ig, immunoglobulin; ANA, antinuclear antibody; C3, complement component 3; C4, complement component 4; WBC, white blood cell; Hb, haemoglobin; PLT, platelet; HAMD, Hamilton Depression Scale; AIDs, autoimmune diseases; ALN, abdominal lymph node.

**Table S2** | Comparison of general characteristics and laboratory parameters between AIH patients with and without depression.

|  | **Without depression**  **(n = 154)** | **With depression**  **(n = 106)** | ***p-*value** |
| --- | --- | --- | --- |
| **General characteristics** |  |  |  |
| Age (median with IQR) | 58.5 (48.0-65.0) | 57.5 (49.0-63.0) | 0.509^†^ |
| Women (n, %) | 131 (85.1%) | 94 (88.7%) | 0.401^‡^ |
| **Laboratory parameters** |  |  |  |
| TP (mean ± SD, g/L) | 74.7 ± 5.1 | 74.4 ± 6.3 | 0.688^§^ |
| ALB (median with IQR, g/L) | 42.0 (40.0-44.0) | 41.0 (40.0-44.0) | 0.054^†^ |
| GLO (median with IQR, g/L) | 32.0 (30.0-35.0) | 32.0 (29.8-36.0) | 0.593^†^ |
| ALT (median with IQR, U/L) | 31.0 (27.0-36.0) | 36.5 (27.0-51.0) | 0.002^†^ |
| AST (median with IQR, U/L) | 33.0 (28.0-38.0) | 37.5 (27.0-50.0) | 0.005^†^ |
| ALP (median with IQR, U/L) | 95.7 (76.0-124.0) | 99.0 (83.0-119.3) | 0.443^†^ |
| GGT (median with IQR, U/L) | 34.0 (22.0-41.3) | 37.0 (23.8-46.3) | 0.069^†^ |
| TB (mean ± SD, μmol/L) | 12.8 ± 2.8 | 13.2 ± 3.3 | 0.374^§^ |
| DB (median with IQR, μmol/L) | 3.6 (3.0-4.5) | 3.7 (2.9-4.6) | 0.459^†^ |
| IgG (median with IQR, mg/dL) | 1450.0 (1320.0-1530.0) | 1610.0 (1400.0-1752.5) | <0.001^†^ |
| IgM (median with IQR, mg/dL) | 121.0 (92.7-165.8) | 128.5 (96.3-169.8) | 0.720^†^ |
| ANA titer (*≥*1:160) (n, %) | 78 (50.6%) | 60 (56.6%) | 0.344^‡^ |
| C3 (median with IQR, mg/dL) | 100.0 (85.5-116.3) | 97.8 (79.0-109.3) | 0.044^†^ |
| C4 (median with IQR, mg/dL) | 21.0 (17.4-25.9) | 19.5 (15.8-21.9) | 0.003^†^ |
| WBC (median with IQR, *10^9^/L) | 5.2 (4.4-6.1) | 5.2 (4.3-6.1) | 0.716^†^ |
| Hb (mean ± SD, g/L) | 131.6 ± 13.4 | 129.9 ± 14.8 | 0.330^§^ |
| PLT (median with IQR, *10^9^/L) | 192.5 (155.0-218.5) | 184.0 (142.8-220.3) | 0.722^†^ |
| **Disease assessment** |  |  |  |
| Cirrhosis (n, %) | 35 (22.7%) | 55 (51.9%) | <0.001^‡^ |
| Extrahepatic AIDs (n, %) | 47 (30.5%) | 73 (68.9%) | <0.001^‡^ |
| Enlarged ALN (n, %) | 68 (44.2%) | 67 (63.2%) | 0.003^‡^ |

Note: Without depression: HAMD score < 8; With depression: HAMD score ≥ 8. ^†^: the statistic value was based on Mann-Whitney U test; ^‡^: the statistic value was based on chi-square test. ^§^: the statistic value was based on unpaired t-test.

Abbreviations: AIH, autoimmune hepatitis; IQR, interquartile range; SD, standard deviation; TP, total protein; ALB, albumin; GLO, globulin; ALT, alanine aminotransferase; AST, aspartate aminotransferase; ALP, alkaline phosphatase; GGT, γ-glutamyl transpeptidase; TB, total bilirubin; DB, direct bilirubin; Ig, immunoglobulin; ANA, antinuclear antibody; C3, complement component 3; C4, complement component 4; WBC, white blood cell; Hb, haemoglobin; PLT, platelet; AIDs, autoimmune diseases; ALN, abdominal lymph node.

**Table S3** | Comparison of general characteristics and laboratory parameters between AIH patients with and without cirrhosis.

|  | **Without cirrhosis**  **(n = 170)** | **With cirrhosis**  **(n = 90)** | ***p-*value** |
| --- | --- | --- | --- |
| **General characteristics** |  |  |  |
| Age (median with IQR) | 56.0 (47.0-63.0) | 61.0 (50.8-66.0) | 0.006^†^ |
| Women (n, %) | 145 (85.3%) | 80 (88.9%) | 0.419^‡^ |
| **Laboratory parameters** |  |  |  |
| TP (mean ± SD, g/L) | 74.3 ± 4.7 | 75.0 ± 7.0 | 0.410^§^ |
| ALB (median with IQR, g/L) | 42.0 (40.0-44.0) | 41.0 (39.0-44.0) | 0.009^†^ |
| GLO (median with IQR, g/L) | 32.0 (30.0-34.3) | 34.0 (29.0-38.0) | 0.017^†^ |
| ALT (median with IQR, U/L) | 31.0 (27.0-36.0) | 38.0 (26.8-51.5) | 0.006^†^ |
| AST (median with IQR, U/L) | 33.0 (28.0-38.0) | 41.0 (31.0-50.5) | <0.001^†^ |
| ALP (median with IQR, U/L) | 93.0 (78.0-110.5) | 110.5 (82.5-136.3) | 0.007^†^ |
| GGT (median with IQR, U/L) | 34.0 (22.0-42.0) | 37.0 (22.5-46.3) | 0.125^†^ |
| TB (mean ± SD, μmol/L) | 12.8 ± 2.7 | 13.2 ± 3.5 | 0.342^§^ |
| DB (median with IQR, μmol/L) | 3.5 (2.9-4.4) | 3.9 (3.1-4.6) | 0.157^†^ |
| IgG (median with IQR, mg/dL) | 1450.0 (1320.0-1530.0) | 1635.0 (1417.5-1732.5) | <0.001^†^ |
| IgM (median with IQR, mg/dL) | 121.0 (91.2-163.0) | 136.0 (103.8-172.8) | 0.252^†^ |
| ANA titer (*≥*1:160) (n, %) | 77 (45.3%) | 61 (67.8%) | 0.001^‡^ |
| C3 (median with IQR, mg/dL) | 100.0 (86.2-116.0) | 96.0 (77.8-108.3) | 0.010^†^ |
| C4 (median with IQR, mg/dL) | 21.0 (18.0-25.3) | 18.6 (14.6-23.0) | 0.001^†^ |
| WBC (median with IQR, *10^9^/L) | 5.4 (4.8-6.1) | 4.8 (4.1-6.0) | 0.001^†^ |
| Hb (mean ± SD, g/L) | 133.2 ± 12.5 | 126.5 ± 15.6 | 0.001^§^ |
| PLT (median with IQR, *10^9^/L) | 200.0 (169.8-220.3) | 169.0 (107.5-215.3) | <0.001^†^ |
| **Disease assessment** |  |  |  |
| Depression (n, %) | 51 (30.0%) | 55 (61.1%) | <0.001^‡^ |
| Extrahepatic AIDs (n, %) | 60 (35.3%) | 60 (66.7%) | <0.001^‡^ |
| Enlarged ALN (n, %) | 72 (42.4%) | 63 (70.0%) | <0.001^‡^ |

Note: Without depression: HAMD score <8; With depression: HAMD score ≥ 8. ^†^: the statistic value was based on Mann-Whitney U test; ^‡^: the statistic value was based on chi-square test. ^§^: the statistic value was based on unpaired t-test.

Abbreviations: AIH, autoimmune hepatitis; IQR, interquartile range; SD, standard deviation; TP, total protein; ALB, albumin; GLO, globulin; ALT, alanine aminotransferase; AST, aspartate aminotransferase; ALP, alkaline phosphatase; GGT, γ-glutamyl transpeptidase; TB, total bilirubin; DB, direct bilirubin; Ig, immunoglobulin; ANA, antinuclear antibody; C3, complement component 3; C4, complement component 4; WBC, white blood cell; Hb, haemoglobin; PLT, platelet; AIDs, autoimmune diseases; ALN, abdominal lymph node.

**Table S4** | Clinical characteristics of participants included in the PCR-based analysis of hepatic NLRP3 pathway expression.

| **Participants** | **Gender** | **Age** | **ALT**  **(U/L)** | **AST (U/L)** | **ALP (**U/L**)** | **GGT (**U/L**)** | |
| --- | --- | --- | --- | --- | --- | --- | --- |
| Control 1 | Women | 30 | 10.0 | 10.0 | 44.0 | 11.0 | |
| Control 2 | Women | 49 | 14.0 | 14.0 | 56.0 | 13.0 | |
| Control 3 | Women | 55 | 9.0 | 10.0 | 65.0 | 19.0 | |
| AIH with depression 1 | Women | 58 | 47.0 | 55.0 | 83.0 | 47.0 | |
| AIH with depression 2 | Women | 62 | 21.0 | 32.0 | 100.0 | 40.0 | |
| AIH with depression 3 | Women | 36 | 67.0 | 49.0 | 96.0 | 44.0 | |
| AIH with depression 4 | Women | 34 | 10.0 | 14.0 | 74.0 | 31.0 | |
| AIH with depression 5 | Women | 64 | 108.0 | 120.0 | 99.0 | 38.0 | |
| AIH with depression 6 | Women | 38 | 54.0 | 98.0 | 115.0 | 26.0 | |
| AIH with depression 7 | Men | 34 | 51.0 | 48.0 | 75.0 | 51.0 | |
| AIH without depression 1 | Women | 42 | 30.0 | 32.0 | 65.0 | 25.0 | |
| AIH without depression 2 | Women | 49 | 22.0 | 28.0 | 83.0 | 13.0 | |
| AIH without depression 3 | Women | 52 | 29.0 | 34.0 | 97.0 | 30.3 | |
| AIH without depression 4 | Women | 24 | 33.0 | 26.0 | 73.0 | 34.0 | |
| AIH without depression 5 | Women | 44 | 31.0 | 36.0 | 71.0 | 21.0 | |
| AIH without depression 6 | Women | 70 | 26.0 | 29.0 | 93.0 | | 39.0 |
| AIH without depression 7 | Men | 40 | 24.0 | 29.0 | 70.0 | | 16.0 |

Abbreviations: AIH, autoimmune hepatitis; ALT, alanine aminotransferase; AST, aspartate aminotransferase; ALP, alkaline phosphatase; GGT, γ-glutamyl transpeptidase.
